# Trends in the incidence and DALYs of schizophrenia at the global, regional and national levels: results from the Global Burden of Disease Study 2017

**DOI:** 10.1017/S2045796019000891

**Published:** 2020-01-13

**Authors:** Hairong He, Qingqing Liu, Ning Li, Liyang Guo, Fengjie Gao, Ling Bai, Fan Gao, Jun Lyu

**Affiliations:** 1 Clinical Research Center, The First Affiliated Hospital of Xi'an Jiaotong University, Xi'an, Shaanxi, China; 2 School of Public Health, Xi'an Jiaotong University Health Science Center, Xi'an, Shaanxi, China; 3 Department of Oncology, The First Affiliated Hospital of Xi'an Jiaotong University, Xi'an, Shaanxi, China; 4 Department of Psychiatry, The First Affiliated Hospital of Xi'an Jiaotong University, Xi'an, Shaanxi 710061, China

**Keywords:** DALYs, incidence, schizophrenia

## Abstract

**Aim:**

Schizophrenia is a serious health problem worldwide. This systematic analysis aims to quantify the burden of schizophrenia at the global, regional and national levels using the Global Burden of Disease Study 2017 (GBD 2017).

**Methods:**

We collected detailed information on the number of incidence cases, disability-adjusted life years (DALYs) and age-standardised incidence rate (ASIR) and age-standardised rate of DALYs (ASDR) during 1990–2017 from GBD 2017. The estimated annual percentage changes (EAPCs) in the ASIR and in the ASDR were calculated to quantify the temporal trends in the ASIR and ASDR of schizophrenia.

**Results:**

Globally there were 1.13 million (95% uncertainty interval [UI] = 1.00 to 1.28) incident schizophrenia cases and 12.66 million (95% UI = 9.48 to 15.56) DALYs due to schizophrenia in 2017. The global ASIR decreased slightly from 1990 to 2017 (EAPC = −0.124, 95% UI = −0.114 to −0.135), while the ASDR was stable. The number of incident cases, DALYs, ASIR and ASDR were higher for males than for females. The incident rate and DALYs rate were highest among those aged 20–29 and 30–54 years, respectively. ASIR and ASDR were highest in East Asia in 2017, at 19.66 (95% UI = 17.72 to 22.00) and 205.23 (95% UI = 153.13 to 253.34), respectively. In 2017, the ASIR was highest in countries with a high-moderate sociodemographic index (SDI) and the ASDR was highest in high-SDI countries. We also found that the EAPC in ASDR was negatively correlated with the ASDR in 1990 (*P* = 0.001, *ρ* = −0.23).

**Conclusion:**

The global burden of schizophrenia remains large and continues to increase, thereby increasing the burden on health-care systems. The reported findings should be useful for resource allocation and health services planning for the increasing numbers of patients with schizophrenia in ageing societies.

## Introduction

Schizophrenia is a severe neurodevelopmental psychiatric affliction that typically manifests behaviourally in late adolescence or early adulthood and is characterised by substantial clinical and biological heterogeneity and underlying genetic and pathophysiological mechanisms that mostly pleiotropic (Vita *et al*., 2016; Hemager *et al*., 2018; Hadar *et al*., 2019). The lifetime prevalence of schizophrenia is near 1%, and only 10–15% of patients with schizophrenia are in gainful employment (Dixon, 2017). Patients with schizophrenia have a substantially higher suicide rate, and their mean age at death is 15 years younger than the general population (Hjorthoj *et al*., 2017; Chan *et al*., 2018).

Genome-wide association studies have identified many genomic loci associated with the risk of schizophrenia (Ma *et al*., 2019), little is known about its underlying mechanisms (Druss, 2018). Most research has found it to be associated with dysregulation of the dopamine system (Weinstein *et al*., 2017). Schizophrenia also tends to increase the prevalence and mortality of other diseases, such as breast cancer (Zhuo and Triplett, 2018), cardiovascular diseases (Kugathasan *et al*., 2018) and diabetes (Cui *et al*., 2017). In short, schizophrenia has a very serious impact on both social interactions and family life, and this situation is exacerbated by poor insight into schizophrenia being prevalent across cultures and across the different phases of the illness (Lysaker *et al*., 2018).

Urban life has been proposed as an environmental risk factor for a higher schizophrenia prevalence (Colodro-Conde *et al*., 2018). Kirkbride *et al*. found that both a high population density and poverty were risk factors for schizophrenia (Kirkbride *et al*., 2017). In addition, Hollander *et al*. found that the risk of schizophrenia is higher among refugees than among non-refugee migrants from similar regions of origin and the native-born Swedish population (Hollander *et al*., 2016). These findings indicate that different development levels and environmental backgrounds will affect the incidence of schizophrenia.

The Global Burden of Disease Study 2017 (GBD 2017) provides a tool to quantify the deterioration in the health status resulting from hundreds of diseases, injuries and risk factors across countries, time, age and sex. The aim of GBD 2017 is to improve health-care systems so that such disparities can be eliminated (GBD 2017 Disease and Injury Incidence and Prevalence Collaborators, 2018; GBD 2017 Diet Collaborators, 2019). We used GBD 2017 to investigate the global trends and distribution of schizophrenia by performing a systematic analysis to yield new estimates of the incidence and disability-adjusted life years (DALYs) of schizophrenia from 1990 to 2017 at the global, regional and national levels. We also aimed to determine how these parameters are related to the development level as measured by the human development index (HDI).

## Methods

Data sources for the disease burden of schizophrenia can be explored using the online GHDx (Global Health Data Exchange) data source query tool (http://ghdx.healthdata.org/gbd-results-tool). We obtained the annual incident cases, DALYs number, age-standardised incidence rate (ASIR) and age-standardised rate of DALYs (ASDR) of schizophrenia from 1990 to 2017 according to sex, 21 regions and 195 countries or territories. Based on the sociodemographic index (SDI) of each country at 2017, the 195 countries or territories were divided into five quintiles: low, low-moderate, moderate, high-moderate and high. The general methods used in GBD 2017 are described in detail on the official website (http://www.healthdata.org/gbd/), which also provides the SDI values from 1990 to 2017 at the global, regional and national levels. Meanwhile, the HDIs of all countries were collected from the World Bank. To analyse global trends we also assessed the trends of schizophrenia according to the following age stratification used in GBD 2017: 10–14, 15–19, 20–24, 25–29, 30–34, 35–39, 40–44, 45–49, 50–54, 55–59, 60–64, 65–69, 70–74, 75–79 and >79 years old.

Two measures were used in this study: incidence and DALYs. The estimated annual percentage change (EAPCs) in the ASIR and the ASDR were calculated to quantify the incidence and mortality trends of schizophrenia. The number of incidence and DALYs at the all-age level were also extracted to determine the burden of schizophrenia. The ASIR corresponds to the number of cases per 100 000 persons, while ASDR corresponds to the years lived with disability and years of life lost per 100 000 persons. The ASR was calculated by summing up the products of the age-specific rates (*a_i_*, where *i* is the *i*th age class, and the number of persons (or the weight) (*w_i_*) in the same age subgroup *i* of the selected reference standard population, then dividing the sum of the standard population weights (Liu *et al*., 2019). Age-standardised populations in the GBD were calculated using the GBD world population age standard. The specific explanations about age classes and their weights can be found in the online Supplementary Appendix of published article by GBD 2017 Mortality Collaborators (2018):
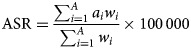


ASR trends can serve as a good surrogate for changing disease patterns in populations and as a clue to changing risk factors. We therefore also evaluated the effectiveness of current prevention strategies based on an analysis of ASRs with the aim of establishing more-targeted approaches.

EAPC was calculated according to Liu *et al*. (2019). EAPC is a summary and widely used measure of ASR trends over a specified time period. The natural logarithm of the regression-line fit to ASR is *y* = *a* + *bx* + *e*, where *x* is the calendar year. EAPC is calculated as 100 × (exp(*b*) − 1), and its 95% confidence interval (CI) can also be obtained from a linear regression model. If the estimated EAPC and the lower bound of its 95% CI are both >0, then ASR is considered to be exhibiting an increasing trend; if both the estimated EAPC and the upper bound of its 95% CI are <0, then ASR is considered to be trending downwards; otherwise ASR is considered stable. In addition, in order to explore the factors influencing EAPC, we evaluated the associations of EAPC with the ASR in 1990 and the HDI in 2017 at the country level using Pearson correlation analysis. The ASR of schizophrenia in 1990 reflects the disease reservoir at baseline and the HDI in 2017 can serve as a surrogate for the quality and availability of health care in each country. All of the analyses were conducted using R program (version 3.5.1, R Core Team).

## Results

### The burden of schizophrenia at the global level

Globally the incident cases and DALYs of schizophrenia increased from 1990 to 2017, in 2017 reaching 1.13 million (95% uncertainty interval [UI] = 1.00 to 1.28 million) and 12.66 million (95% UI = 9.48 to 15.56 million), respectively. Compared with 1990, the incident cases and DALYs had increased by 36.69 and 62.46%, respectively. The incident cases and DALYs in any year were higher for males than for females; in 2017 they were 0.6 million (95% UI = 0.53 to 0.67 million) and 6.51 million (95% UI = 4.86 to 8.02 million), respectively, for males, and 0.54 million (95% UI = 0.48 to 0.61 million) and 6.14 million (95% UI = 4.61 to 7.54 million) for females.

The ASIR decreased slightly from 1990 to 2017 (EAPC = −0.124, 95% UI = −0.114 to −0.135), being 14.98 (95% UI = 13.32 to 16.93) in 1990 and 14.39 (95% UI = 12.78 to 16.3) in 2017 per 100 000 persons. The same global trend was found separately for males (EAPC = −0.123, 95% UI = −0.113 to −0.134) and females (EAPC = −0.125, 95% UI = −0.114 to −0.136), being 15.66 (95% UI = 13.95 to 17.7) in 1990 and 15.04 (95% UI = 13.34 to 17.03) in 2017 per 100 000 persons for males, and 14.3 (95% UI = 12.68 to 16.14) in 1990 and 13.73 (95% UI = 12.19 to 15.59) in 2017 per 100 000 persons for females. The ASIR in any year was higher for males than for females.

The ASDR was stable from 1990 to 2017, being 157.01 (95% UI = 117.53 to 193.51) in 1990 and 156.45 (95% UI = 117.1 to 192.28) in 2017 per 100 000 persons. The ASDR was also stable for males and females separately, being 162.48 (95% UI = 121.47 to 200.61) in 1990 and 161.56 (95% UI = 120.53 to 198.88) in 2017 per 100 000 persons for males, and 151.38 (95% UI = 113.58 to 185.79) in 1990 and 151.28 (95% UI = 113.17 to 185.81) in 2017 per 100 000 persons for females. The ASDR in any year was higher for males than for females. The EAPC in ASDR was low; the values are listed in Table 1.

**Table 1.** The incident cases and DALYs and their age-standardised rate of schizophrenia in 1990 and 2017, and their temporal trends from 1990 to 2017

	Incidence					DALYs				
	Cases in 1990 (No. × 10^3^)	ASR in 1990 (per 100 000 persons)	Cases in 2017 (No. × 10^3^)	ASR in 2017 (per 100 000 persons)	EAPC	Cases in 1990 (No. × 10^4^)	ASR in 1990 (per 100 000 persons)	Cases in 2017 (No. × 10^4^)	ASR in 2017 (per 100 000 persons)	EAPC
Global	827.03 (730.77–939.75)	14.98 (13.32–16.93)	1130.49 (1004.58–1281.9)	14.39 (12.78–16.3)	−0.124 (−0.114 to −0.135)	779.12 (582.92–961.03)	157.01 (117.53–193.51)	1265.79 (948.19–1556.37)	156.45 (117.1–192.28)	−0.005 (−0.009–0)
Sex										
Male	435.97 (385.74–495.06)	15.66 (13.95–17.7)	595.28 (528.52–674.57)	15.04 (13.34–17.03)	−0.123 (−0.111 to −0.134)	405.24 (302.7–500.15)	162.48 (121.47–200.61)	651.49 (485.92–801.9)	161.56 (120.53–198.88)	−0.002 (−0.01–0.007)
Female	391.06 (345.31–445.09)	14.3 (12.68–16.14)	535.21 (476.06–607.34)	13.73 (12.19–15.59)	−0.125 (−0.114 to −0.136)	373.88 (280.16–459.71)	151.38 (113.58–185.79)	614.3 (460.78–754.36)	151.28 (113.17–185.81)	−0.005 (−0.01–0)
Socio-demographic index										
High SDI	144.55 (129.85–162.55)	14.32 (12.83–16.08)	154.54 (139.13–172.69)	14.53 (12.93–16.4)	0.1 (0.117–0.084)	184.48 (139.34–225.68)	168.11 (126.81–206.2)	236.08 (178.79–288.67)	171.25 (128.38–210.75)	0.099 (0.09–0.108)
High-middle SDI	186.18 (167.05–210.86)	15.65 (14.09–17.59)	232.56 (209.73–261.67)	15.76 (14.19–17.72)	0.042 (0.05–0.034)	175.51 (131.91–214.81)	157.28 (118.32–192.7)	284.77 (214.24–348.97)	166.91 (125.25–204.45)	0.209 (0.201–0.218)
Low SDI	83.78 (72.96–96.15)	13.46 (11.84–15.39)	161.43 (141.36–185.39)	12.99 (11.47–14.84)	−0.114 (−0.103 to −0.124)	68.88 (51.18–85.81)	134.28 (100.02–166.1)	136.35 (102.48–169.69)	131.09 (98.2–162.5)	−0.067 (−0.077 to −0.057)
Low-middle SDI	142.95 (124.81–164.04)	14.23 (12.54–16.25)	238.78 (208.99–273.84)	13.61 (12–15.54)	−0.146 (−0.137 to −0.155)	121.32 (90.46–150.52)	144.64 (108.33–178.87)	224.22 (166.28–278.13)	142.12 (105.26–175.66)	−0.046 (−0.055 to −0.037)
Middle SDI	264.49 (231.3–302.62)	16.1 (14.3–18.25)	337.67 (299.38–383.58)	15.17 (13.44–17.25)	−0.214 (−0.211 to −0.218)	224.5 (166.54–276.4)	161.48 (120.43–198.65)	377.61 (280.3–464.98)	160.62 (119.34–197.64)	−0.025 (−0.028 to −0.021)
Region										
Andean Latin America	4.48 (3.9–5.2)	12.23 (10.78–14.02)	7.72 (6.79–8.91)	12.26 (10.83–14.05)	0.004 (0.013 to −0.004)	3.7 (2.73–4.65)	122.69 (91.36–153.44)	7.53 (5.63–9.42)	125.26 (93.77–155.89)	0.074 (0.061–0.087)
Australasia	3.62 (3.19–4.06)	17.04 (14.98–19.17)	4.52 (3.99–5.08)	16.9 (14.74–19.04)	−0.021 (−0.013 to −0.029)	4.55 (3.42–5.61)	205.02 (154.35–252.69)	6.71 (5.03–8.35)	204.92 (153.98–255.17)	0.008 (−0.001–0.016)
Caribbean	4.33 (3.79–4.99)	11.97 (10.55–13.7)	5.7 (5.06–6.5)	11.85 (10.52–13.52)	−0.043 (−0.033 to −0.052)	3.8 (2.84–4.75)	120.14 (90.06–148.88)	5.92 (4.42–7.34)	119.96 (89.44–148.78)	0.007 (0–0.014)
Central Asia	7.47 (6.48–8.73)	10.66 (9.38–12.32)	10.49 (9.17–12.23)	10.72 (9.41–12.4)	0.021 (0.035–0.006)	6.97 (5.19–8.68)	115.61 (86.26–143.35)	11.12 (8.25–13.94)	117.4 (87.4–147.09)	0.065 (0.025–0.105)
Central Europe	13.73 (12.11–15.74)	11.13 (9.76–12.88)	12.21 (10.78–13.93)	11.26 (9.84–13)	0.044 (0.049–0.039)	16.32 (12.21–20.24)	119.6 (89.47–148.62)	17.43 (13.03–21.59)	123.01 (92–152.14)	0.112 (0.106–0.118)
Central Latin America	19.77 (17.2–22.96)	12.36 (10.91–14.17)	33.06 (28.98–38.01)	12.36 (10.87–14.15)	−0.007 (−0.005 to −0.009)	16.21 (12–20.33)	125.47 (93.19–155.78)	32.85 (24.49–40.6)	126.85 (94.58–156.52)	0.028 (0.024–0.032)
Central Sub-Saharan Africa	5.49 (4.78–6.34)	11.65 (10.31–13.29)	12.67 (11.11–14.52)	11.63 (10.29–13.21)	−0.009 (0.002 to −0.02)	3.81 (2.81–4.81)	99.09 (73.49–124.01)	9.03 (6.7–11.36)	99.77 (74.42–124.44)	0.055 (0.006–0.104)
East Asia	282.31 (252.28–318.44)	19.93 (17.97–22.29)	304.92 (275.35–341.91)	19.66 (17.72–22)	−0.059 (−0.051 to −0.066)	245.49 (183.96–301.84)	197.08 (148.01–241.38)	382.25 (286.61–468.75)	205.23 (153.13–253.34)	0.134 (0.115–0.153)
Eastern Europe	23.77 (21–27.27)	10.37 (9.13–11.97)	21.59 (19.12–24.73)	10.55 (9.3–12.18)	0.089 (0.127–0.051)	29.21 (21.91–36.2)	114.04 (85.76–142.27)	30.42 (22.79–37.53)	116.48 (86.83–144.22)	0.112 (0.057–0.167)
Eastern Sub-Saharan Africa	18.75 (16.47–21.53)	11.8 (10.5–13.35)	41.6 (36.48–47.7)	11.8 (10.51–13.39)	−0.002 (0.005 to −0.009)	12.59 (9.35–15.81)	98.28 (73.6–122.16)	28.86 (21.53–36.12)	101.85 (76.52–126.34)	0.151 (0.121–0.181)
High-income Asia Pacific	26.66 (23.51–30.38)	14.51 (12.78–16.69)	24.81 (22.03–27.86)	14.72 (12.95–16.93)	0.13 (0.164–0.095)	32.95 (24.43–40.67)	165.63 (122.56–204.74)	38.64 (28.92–47.41)	166.21 (124.33–205.22)	0.095 (0.06–0.131)
High-income North America	42.32 (37.69–47.93)	14.53 (12.93–16.38)	47.1 (42.16–52.66)	14.16 (12.63–15.95)	−0.06 (−0.03 to −0.091)	61.22 (46.08–75.17)	195.35 (147.1–239.79)	79.7 (60.09–97.26)	190.82 (143.29–233.16)	−0.034 (−0.065 to −0.003)
North Africa and Middle East	38.31 (33.44–44.06)	11.9 (10.5–13.6)	75.42 (65.95–86.74)	11.82 (10.4–13.5)	−0.007 (0 to −0.014)	30.62 (22.58–37.89)	116.48 (86–142.97)	71.15 (53.07–88.21)	118.47 (88.13–146.38)	0.079 (0.073–0.085)
Oceania	0.91 (0.79–1.05)	14.59 (12.89–16.61)	1.85 (1.62–2.15)	14.56 (12.87–16.71)	−0.01 (−0.003 to −0.017)	0.68 (0.5–0.85)	132.11 (97.45–164.01)	1.47 (1.09–1.85)	132.5 (98.76–165.21)	−0.017 (−0.032 to −0.003)
South Asia	162.16 (140.74–186.84)	14.92 (13.07–17.06)	274.57 (239.02–314.67)	14.41 (12.65–16.49)	−0.085 (−0.059 to −0.112)	148.84 (111.41–184.39)	162.44 (122.02–200.97)	278.81 (208.24–345.21)	160.04 (119.72–198.08)	−0.013 (−0.034–0.007)
Southeast Asia	67.05 (58.3–77.37)	14.2 (12.54–16.15)	102.87 (90.75–117.04)	14.63 (12.9–16.66)	0.106 (0.111–0.102)	53.82 (39.75–67.21)	134.46 (99.64–166.68)	102.19 (76.44–126.35)	144.23 (108.05–178.27)	0.246 (0.238–0.253)
Southern Latin America	5.24 (4.58–5.96)	10.67 (9.31–12.13)	7.18 (6.31–8.14)	10.58 (9.28–12.02)	−0.012 (−0.002 to −0.021)	5.59 (4.17–6.95)	116.51 (86.84–144.78)	8.39 (6.3–10.44)	116.75 (87.19–145.78)	0.027 (0.021–0.034)
Southern Sub-Saharan Africa	5.95 (5.19–6.85)	11.81 (10.42–13.42)	9.63 (8.46–11.09)	11.79 (10.45–13.35)	−0.006 (0 to −0.012)	4.46 (3.3–5.57)	107.38 (80.6–133.4)	8.09 (6.1–10.12)	107.72 (81.54–133.92)	0.002 (−0.022–0.026)
Tropical Latin America	19.3 (16.83–22.34)	12.43 (10.94–14.2)	29.49 (26.04–33.82)	12.45 (10.97–14.29)	0 (0.005 to −0.006)	16.43 (12.34–20.47)	123.53 (92.51–152.58)	30.77 (22.98–38.03)	125.5 (93.83–154.81)	0.052 (0.039–0.065)
Western Europe	55.31 (50.31–61.32)	13.78 (12.48–15.32)	55.9 (50.68–61.81)	13.82 (12.45–15.49)	−0.008 (−0.003 to −0.014)	67.41 (51.14–81.55)	150.64 (113.42–183.06)	80.13 (60.84–97.32)	151.72 (115.4–185.53)	0.011 (0.006–0.016)
Western Sub-Saharan Africa	20.1 (17.66–23.09)	12.18 (10.78–13.75)	47.19 (41.35–54.17)	12.22 (10.85–13.8)	0.014 (0.021–0.008)	14.47 (10.64–18.02)	105.89 (78.39–131.13)	34.32 (25.57–42.92)	109.11 (81.72–135.43)	0.134 (0.106–0.162)

**Fig. 1.**
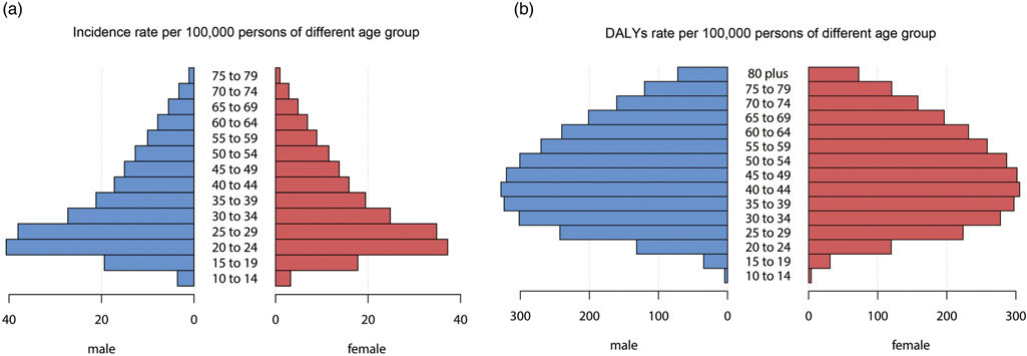
The incident rate (*a*) and DALYs rate (*b*) of schizophrenia of different age group globally. DALYs, disability-adjusted life years.

We also analysed the incident rate and DALYs rate for males and females in the different age groups. The incident rate and DALYs rate were higher for males than for females in all age groups except for DALYs rate among those aged ⩾75 years, for which the values were mostly the same. The incident rate was highest in those aged 20–29 years, while the DALYs rate were highest among those aged 30–34, 35–39, 40–44, 45–49 and 50–54 years (Fig. 1).

### The burden of schizophrenia at the national level

Among the 20 most-populous countries globally, the absolute numbers of newly diagnosed schizophrenia cases in 2017 were largest in China (0.29 million, 95% UI = 0.26 to 0.33 million), India (0.21 million, 95% UI = 0.19 to 0.25 million) and the United States (42 883.34, 95% UI = 37 907.73 to 48 469.29), and the smallest in the Democratic Republic of the Congo (8385.79, 95% UI = 7334.14 to 9581.22). The DALYs in 2017 was highest in China (3.67 million, 95% UI = 2.75 to 4.5 million), followed by India (2.21 million, 95% UI = 1.67 to 2.74 million) and the United States (0.72 million, 95% UI = 0.54 to 0.88 million), and lowest in the Democratic Republic of the Congo (58 548.38, 95% UI = 43 296.65 to 74 579.92).

**Fig. 2.**
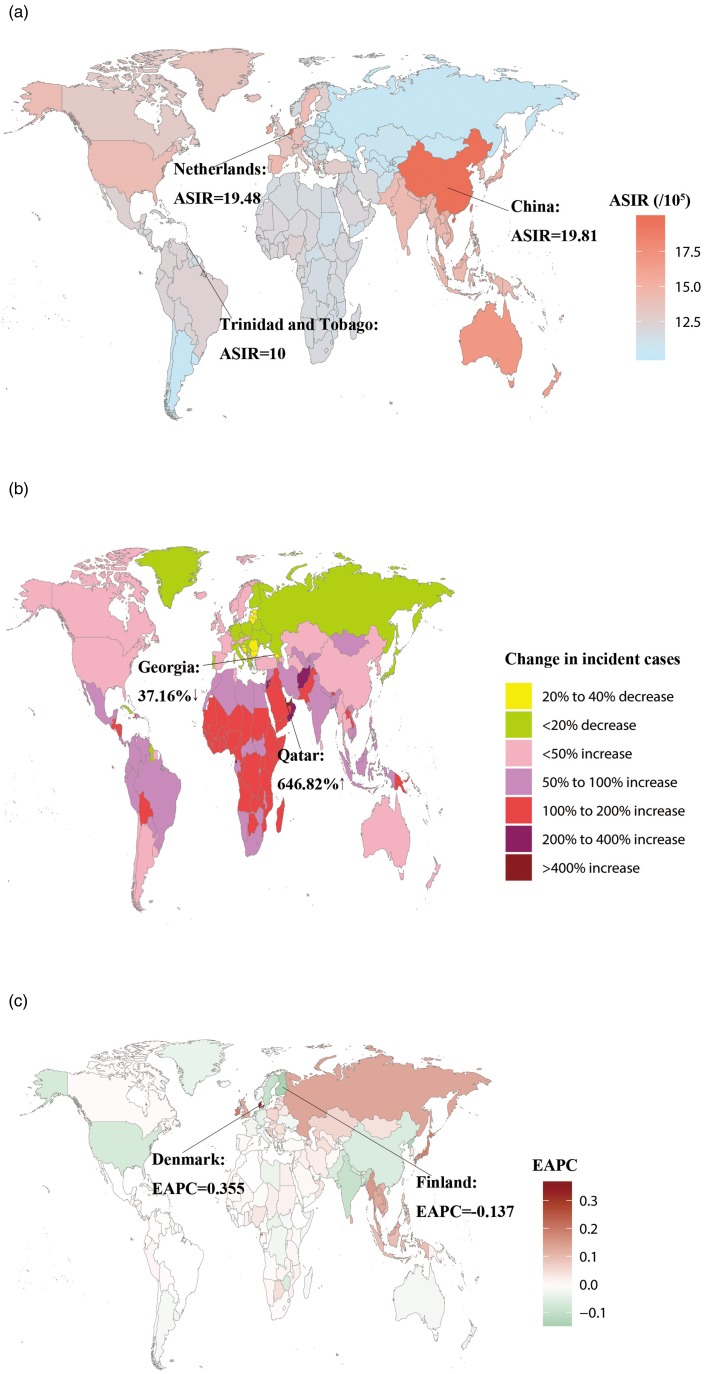
The global disease burden of schizophrenia in 195 countries or territories. (*a*) The ASIR of schizophrenia in 2017. (*b*) The relative change in incident cases of schizophrenia between 1990 and 2017. (*c*) The EAPC of ASIR of schizophrenia from 1990 to 2017. Countries with an extreme number of cases/evolution were annotated. ASIR, age-standardised incident rate; EAPC, estimated annual percentage change.

Online Supplementary Table 1 lists the incident cases and DALYs and their age-standardised rate of schizophrenia of 195 countries and territories in 1990 and 2017. Compared with the incidence and DALYs number in 1990, the increase by 2017 was most pronounced in Qatar and the United Arab Emirates (646.82 and 477.57% for incidence; 682.74 and 678.02% for DALYs, respectively). The number of incidence decreased over time in most European countries, with the largest decrease in Georgia (37.16 and 27.1% for incidence and DALYs, respectively) (Fig. 2*b*, online Supplementary Fig. 1B).

The ASIR of schizophrenia varied slightly globally, being highest in 2017 in China (19.81 [95% UI = 17.87 to 22.14] per 100 000 persons), followed by Netherlands and Australia, and lowest in Trinidad and Tobago (The value is about 10 per 100 000 persons) (Fig. 2*a*). The ASIR was approximately stable over time in most countries, with only slightly increases or decreases. The clearest increasing trend was in Denmark (EAPC = 0.355, 95% UI = 0.265 to 0.444) and the most-significant decrease was in Finland (EAPC = −0.137, 95% UI = −0.086 to −0.189) (Fig. 2*c*).

The ASDR in 2017 varied markedly worldwide, being highest in Netherlands (207.99 [95% UI = 155.68 to 255.34] per 100 000 persons) followed by Australia and China, and the lowest in Central African Republic (92.21 [95% UI = 68.2 to 114.57] per 100 000 persons) (online Supplementary Fig. 1A). The temporal trend in ASDR differed between countries, with Equatorial Guinea, Myanmar, Maldives, Denmark, Cambodia, Laos and Timor-Leste showing obvious increasing trends (EAPC > 0.3). The increasing trend was largest in Equatorial Guinea (EAPC = 0.83, 95% UI = 0.71 to 0.95) while the most-significant decreasing trend was in North Korea (EAPC = −0.227, 95% UI = −0.182 to −0.272) (online Supplementary Fig. 1C).

### The burden of schizophrenia at the regional level

**Fig. 3.**
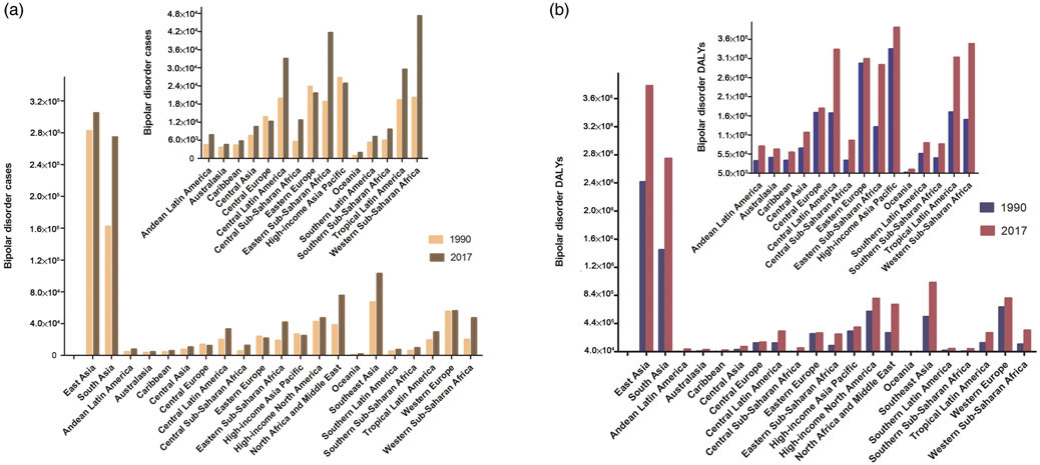
The incident cases and DALYs of schizophrenia at a regional level. The left column in each group is case data in 1990 and the right column in 2017. DALYs, disability-adjusted life years.

The number of incidence and DALYs differed among the 21 regions (Fig. 3). Overall, the incident cases and DALYs in 2017 were highest in East Asia (304 918.2, 95% UI = 275 346.9 to 341 906.3; and 3.82 million, 95% UI = 2.87 to 4.69 million; respectively) and South Asia (274 565.3, 95% UI = 239 023.1 to 314 673.3; and 2.79 million, 95% UI = 2.08 to 3.45 million; respectively) and lowest in Oceania (1850.69, 95% UI = 1616.97 to 2146.14; and 14 741.76, 95% UI = 10 923.77 to 18 500.82; respectively). Both indexes were lower in 1990 than in 2017 in all regions with the exceptions of Central Europe, high-income Asia-Pacific and Eastern Europe, in which the incident cases was higher in 1990 than in 2017. In addition, the incident number in Western Europe was almost the same in 1990 and 2017. Compared with 1990, Central Sub-Saharan Africa and Western Sub-Saharan Africa showed the largest increases in 2017 in the incident cases (130.73 and 134.77%, respectively) and DALYs (137.03 and 137.19%, respectively).

**Fig. 4.**
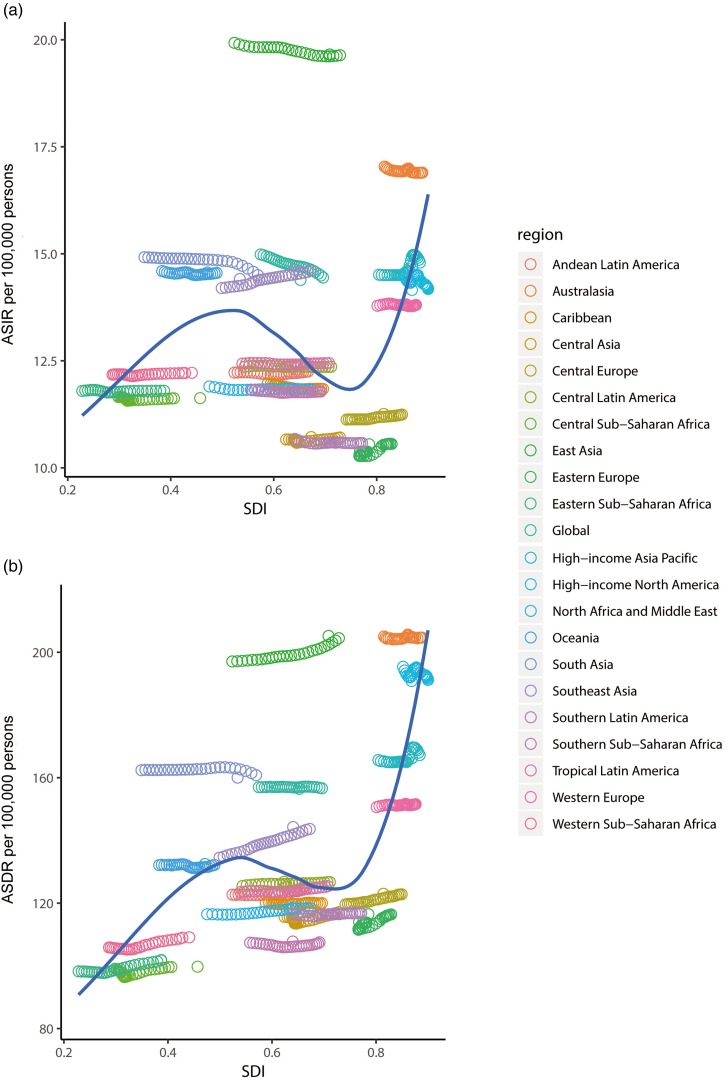
ASIR (*a*) and ASDR (*b*) for schizophrenia by SDI, 1990–2017, and expected value-based SDI. The black line represents the average expected relationship between SDI and incidence (*a*) or DALYs (*b*) for schizophrenia based on values from all countries over the 1990–2017 estimation period. ASIR, age-standardised incident rate; ASDR, age-standardised rate of DALYs; SDI, sociodemographic index; DALYs, disability-adjusted life-years.

Figure 4 shows the SDI and ASR in the 21 regions from 1990 to 2017. The SDI increased in all 21 regions over the 28-year period. As shown in Fig. 4*a*, the ASIR first increased in moderate-SDI countries, and then decreased for regions with SDI values from 0.52 to 0.78 before exhibiting another rapid increase. In most regions the ASIR was stable over time, with only slightly increases or decreases (Table 1). The ASIR in 2017 was highest in East Asia (19.66 [95% UI = 17.72 to 22] per 100 000 persons) and lowest in Eastern Europe (10.55 [95% UI = 9.30 to 12.18] per 100 000 persons). Figure 4*b* shows that the ASDR mostly increased with the SDI, with only slight decreased at SDI values from 0.52 to 0.70. The ASDR in almost all regions were also relatively stable, with only slightly increases or decreases (Table 1), but in Southeast Asia the ASDR showed a clear upward trend (EAPC = 0.246, 95% UI = 0.238 to 0.253). The ASDR in 2017 was highest in East Asia (205.23 [95% UI = 153.13 to 253.34] per 100 000 persons) and Australasia (204.92 [95% UI = 153.98 to 255.17] per 100 000 persons), and lowest in Central Sub-Saharan Africa (99.77 [95% UI = 74.42 to 124.44] per 100 000 persons).

### The burden of schizophrenia at the SDI-quintile level

**Fig. 5.**
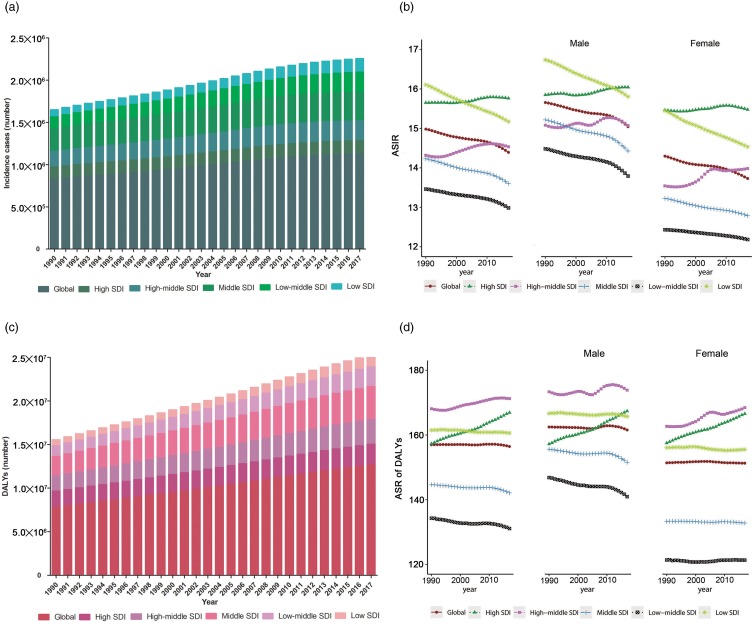
The incident cases (*a*), ASIR (*b*), DALYs number (*c*) and ASDR (*d*) for schizophrenia from 1990 to 2017 at SDI quintiles level. ASIR, age-standardised incident rate; ASDR, age-standardised rate of DALYs; DALYs, disability-adjusted life-years; SDI, sociodemographic index.

The incident cases and DALYs showed slightly increasing trends in all SDI-quintile countries. The value in any year was higher in moderate-SDI countries (Fig. 5*a*, *c*). The ASIR and ASDR showed slight upward trends in high-SDI and high-moderate-SDI countries (EAPC > 0), and downward trends in the other countries (EAPC < 0). The ASIR was highest in moderate-SDI countries in 1990, while in high-middle SDI quintile in 2017 (15.76 [95% UI = 14.19 to 17.72] per 100 000 persons), and it was lowest in low-SDI countries in any year, in which it was 12.99 (95% UI = 11.47 to 14.84) in 2017 per 100 000 persons. The ASDR was highest in 2017 in high-SDI countries (171.25 [95% UI = 128.38 to 210.75] per 100 000 persons) and lowest in any year in low-SDI countries (131.09 [95% UI = 98.2 to 162.5] per 100 000 persons). The ASIR was higher in high-moderate-SDI and moderate-SDI countries than the global average, and lower in low-SDI and low-moderate-SDI countries. The ASIR in high-SDI countries was lower than the global average before 2012 and then increased to above the global average. The ASDR was higher in high-SDI, high-moderate-SDI and moderate-SDI countries than the global average, and lower in low-SDI and low-moderate SDI countries than the global average (Fig. 5*b*, *d*).

The trends for males and females among the five SDI quintiles were basically the same as those for the total cohort. Both ASIR and the ASDR were higher for males than for females in any SDI quintile in any year. In addition, the ASDR for males in high-middle SDI quintile was lower than that for all males before 2006 (Fig. 5*b*, *d*).

### Relationship between EAPC and economic health indicators

**Fig. 6.**
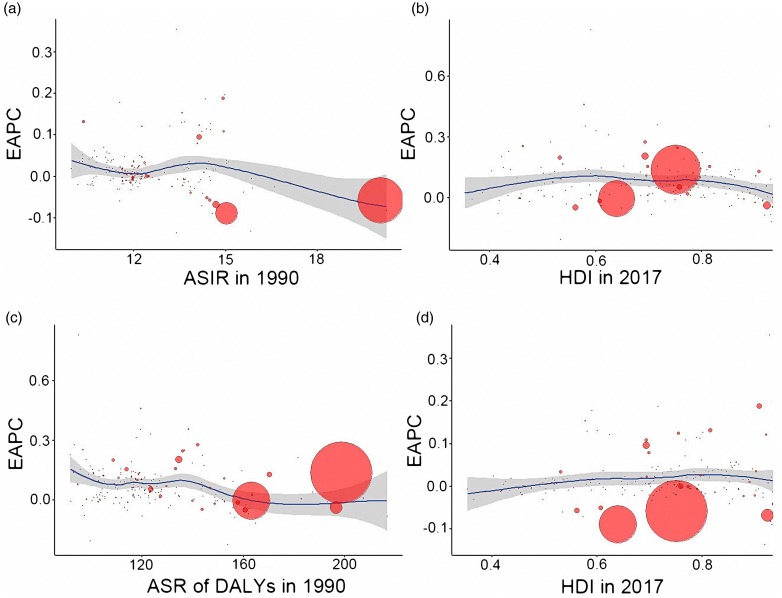
The correlation between EAPC of ASIR and ASIR in 1990 (*a*); EAPC of ASIR and HDI in 2017 (*b*); EAPC of ASDR and ASDR in 1990 (*c*); EAPC of ASDR and HDI in 2017 (*d*) of 195 countries or territories. The size of the circle represents the number of cases in this country in 2017. ASIR, age-standardised incident rate; ASDR, age-standardised rate of DALYs; EAPC, estimated annual percentage change; HDI, human development index; DALYs, disability-adjusted life years.

We analysed the relationships between EAPCs and ASRs in 1990 and HDI in 2017 for the 195 included countries or territories. The analysis revealed a negative correlation between the EAPC in ASDR and the ASDR in 1990 (*p* = 0.001, *ρ* = −0.23). No significant association was found between the EAPCs in ASIR and ASIR in 1990. The HDI in 2017 did not seem to affect the trends in the ASIR or ASDR (Fig. 6).

## Discussion

Schizophrenia has a large global burden. The DALYs caused by schizophrenia in females ranked 19th among the all-causes DALYs in females globally in 2017 (GBD 2017 Disease and Injury Incidence and Prevalence Collaborators, 2018). We found that 19.78 million persons were suffering schizophrenia in 2017, which represented an increase of 62.74% compared with 1990. In 2017, the DALYs caused by schizophrenia accounted for 0.51% of the all-cause DALYs. We estimate that the actual burden of schizophrenia may be higher because the quality of care remains inadequate – even in wealthier countries – due to schizophrenia continuing to be a stigmatised and poorly understood condition (Barnett, 2018).

The ASIR of schizophrenia was higher in males than in females, which differs from the sex-related rates for depression and anxiety disorders, for which the ASIR in males are lower than that in females by querying the GBD 2017 database. The ASIR and ASDR also varied in the different age groups in this study, with ASIR being higher among young people, which is consistent with the characteristics of schizophrenia, while both young and middle-aged people contributed to higher DALYs.

Globally, schizophrenia is more common in East Asia and South Asia, especially in China and India. In 2017, the DALYs in China and India accounted for 35% of the total DALYs caused by schizophrenia, with new cases accounting for 45%. The incidence number for schizophrenia in most parts of Africa, West Asia and South America has increased significantly over the past 28 years, which is related to differences in health conditions and population growth rates. In Qatar and the United Arab Emirates the incidence number had increased in 2017 by four- to seven-fold compared with the values in 1990, which is mainly due to the populations of these two countries surging by 470 and 339%, respectively. The decreases in the incidence number for schizophrenia in Russia and its neighbouring European countries, Japan and Greenland are analogously mainly due to decreasing populations. Compared with 1990, the basic DALYs of each country had increased in 2017, indicating that the disease burden of schizophrenia has not been alleviated.

While the incidence number of schizophrenia and the resulting DALYs are high, the ASIR of schizophrenia globally and the ASDR have not changed significantly, with the ASIR even slightly decreasing. The ASIR and ASDR in 2017 differed markedly between different countries and regions. East Asia and Australasia exhibited the highest ASIR and ASDR, mainly due to contributions from China and Australia. In addition to the large number of cases, China had the highest ASIR and ASDR in 2017, and hence the disease burden caused by schizophrenia is greater in China. It is worth noting that although the ASIRs of China, India, Australia and the United States are relatively high, they had not increased much or have even slightly decreased over the 28-year analysis period, while in Russia, although the ASIR is low, there has been a growth trend over the same period. The ASIR of North America (including the United States and Canada) and Greenland in 2017 is not very high, but their ASDR were high, which might be mainly attributable to the longer life expectancy in these high-SDI countries.

It is puzzling that the ASIR of schizophrenia and ASDR did not increase monotonically with the SDI at the regional level, instead showing an N-shaped trend. The ASIR was higher in the moderate-SDI and high-moderate-SDI regions than the overall global average, and lower in the low-SDI and low-moderate-SDI regions. The ASIR in 1990 was highest in the moderate-SDI countries, and had increased slightly 28 years later in the high-moderate SDI countries. In contrast, the ASIR decreased slightly in the moderate-SDI countries, resulting in it being highest in 2017 in high-moderate-SDI countries. The ASIR in high-SDI countries was close to the global average, while the ASDR were the highest in those countries. This appears to contradict the argument that poverty is a risk factor for schizophrenia. However, we speculate that young people in the higher-moderate-SDI and moderate-SDI regions face greater social pressures. In addition, these two groups of regions have higher levels of urbanisation, which is also a risk factor for schizophrenia (Colodro-Conde *et al*., 2018). The socioeconomic level is generally low in low-SDI regions, with less urbanisation, which may lead to a lower incidence of schizophrenia. Of course, we cannot rule out countries and families with lower socioeconomic levels paying less attention to schizophrenia, leading to more patients not being diagnosed in time. In high-SDI countries, although the abundance of health-care resources controls the incidence of schizophrenia, the increased life expectancy increases the loss of DALYs.

The current status of treatment of schizophrenia remains pessimistic (Isohanni *et al*., 2018), and early discovery is very important for the prevention and treatment of diseases. The overwhelming human and economic repercussions of mental illnesses make mental health reforms a worldwide priority. Schizophrenia is not without precursors. Specific motor abnormalities related to fine motor function and balance first appear in children with a familial risk of schizophrenia at 7 years of age. Childhood cognitive, social, behavioural and emotional impairments are implicated as antecedents to a high risk of schizophrenia (Riglin *et al*., 2017). In addition, a long duration of untreated psychosis and substance misuse at baseline were found to be significant predictors of mortality (Melle *et al*., 2017). Early-intervention services for people with psychosis are already well established in East Anglia (UK) (Kirkbride *et al*., 2017), and they may be able to provide some guidance. In addition, schizophrenia is strongly heritable, and so a focus on children and families at increased risk of schizophrenia or mood disorders will be central to providing the necessary social support and medical services.

The limitations of this study are mainly related to the limitations of the GBD 2017 database itself, which have already been reported in many articles (GBD 2015 Disease and Injury Incidence and Prevalence Collaborators, 2016; GBD 2017 Causes of Death Collaborators, 2018; GBD 2017 DALYs and HALE Collaborators, 2018; GBD 2017 Risk Factor Collaborators, 2018). Briefly, data of GBD are calculated using an algorithm based on existing data in every country, that is, the accuracy of GBD data depends largely on the quality and quantity of data used in the algorithm. Therefore, the data of countries with low level of development have great limitations, and the conclusion about country with the highest/lowest disease level specified in this article should be treated with caution. In addition, a large proportion of the serious social burden is caused by refractory schizophrenia, for which drug treatment is ineffective. These patients should receive more attention, but the disease classifications in the GBD 2017 database made it impossible to understand the epidemiological situation of this part of the population.

The present study used the GBD 2017 database to describe the disease burden of schizophrenia in various countries and regions around the world. The results could be significant in guiding the formulation of suitable medical and economic policies at both the country and region levels. There has been no significant change in the ASIR of schizophrenia globally, and the absolute number of cases and DALYs in 2017 remain high – the large burden of schizophrenia needs attention. Therefore, family concerns and social support are important for controlling the incidence of schizophrenia and the burden of disease. The provision of appropriate medical services needs to be improved, especially in densely populated regions of the world.

## Supplementary material

For supplementary material accompanying this paper visit http://dx.doi.org/10.1017/S2045796019000891. click here to view supplementary material

## Data

The data that support the findings of this study are available from the corresponding author upon reasonable request.
