# Supplementary material for: Trends in the incidence and DALYs of schizophrenia at the global, regional and national levels: results from the Global Burden of Disease Study 2017
Source: Epidemiol Psychiatr Sci. 2020 Jan 13;29:e91. doi: 10.1017/S2045796019000891 (PMC7214712; doi:10.1017/S2045796019000891)
Supplement: Supplementary file 1 [file S2045796019000891sup001.pdf]

Trends in the incidence and DALYs of schizophrenia at the global, regional, and national levels: results from the Global Burden of Disease Study 2017

Contents

Table S1. The incident cases and age-standardized incidence of bipolar disorder in 1990 and 2017, and its temporal trends from 1990 to 2017 at national level.

Figure S1. The global disease burden of bipolar disorder in 195countries or territories. (A) The ASDR of schizophrenia in 2017; (B) The relative change in DALYs number caused by schizophrenia between 1990 and 2017; (C) The EAPC of ASDR of schizophrenia from 1990 to 2017.Countries with an extreme number of cases/evolution were annotated. ASIR, age-standardized incident rate; ASDR, age-standardized rate of DALYs; EAPC, estimated annual percentage change; DALYs, disability-adjusted life years.

Table S1. The incident cases and age-standardized incidence of bipolar disorder in 1990 and 2017, and its temporal trends from 1990 to 2017 at national level.

Country	Incidence					DALY				
	Cases in 1990 (No.×10²)	ASR in 1990 (per 100,000 persons)	Cases in 2017 (No.×10²)	ASR in 2017 (per 100,000 persons)	EAPC	Number in 1990 (No.×10²)	ASR in 1990 (per 100,000 persons)	Number in2017 (No.×10²)	ASR in 2017 (per 100,000 persons)	EAPC
Afghanistan	8.9 (7.7 - 10.11)	11.11 (9.79 - 12.65)	32.36 (27.79 - 37.56)	11.08 (9.74 - 12.67)	0.008 (0 - 0.016)	68.09 (50.7 - 84.22)	95.81 (72.42 - 119.43)	217.97 (159.66 - 274.47)	99.13 (74.23 - 123.39)	0.15 (0.137 - 0.162)
Albania	3.8 (3.29 - 4.43)	11.12 (9.73 - 12.79)	3.15 (2.75 - 3.62)	11.21 (9.78 - 13.02)	0.033 (0.031 - 0.035)	34.9 (25.52 - 43.48)	118.83 (87.48 - 147.49)	38.49 (28.35 - 47.81)	122.19 (88.94 - 152.77)	0.123 (0.114 - 0.133)
Algeria	28.11 (24.24 - 32.89)	11.79 (10.37 - 13.49)	51.7 (45.02 - 59.64)	11.76 (10.27 - 13.47)	0.002 (-0.003 - 0.008)	217.84 (160.68 - 275.69)	117.46 (86.81 - 147.53)	505.97 (371.12 - 634.37)	117.98 (86.93 - 147.29)	0.048 (0.037 - 0.059)
American Samoa	0.08 (0.07 - 0.09)	15.61 (13.72 - 17.99)	0.09 (0.07 - 0.1)	15.38 (13.52 - 17.7)	-0.039 (-0.045 - -0.032)	0.62 (0.45 - 0.78)	154.96 (114.49 - 194.16)	0.78 (0.58 - 0.98)	151.13 (110.92 - 188.58)	-0.068 (-0.081 - -0.056)
Andorra	0.09 (0.08 - 0.11)	14 (12.23 - 16.08)	0.11 (0.1 - 0.13)	13.87 (12.05 - 15.92)	-0.031 (-0.038 - -0.024)	1.02 (0.76 - 1.29)	156 (115.94 - 196.12)	1.66 (1.23 - 2.05)	153.96 (113.4 - 192.38)	-0.041 (-0.051 - -0.031)
Angola	10.51 (9.09 - 12.12)	11.75 (10.33 - 13.39)	28.52 (24.84 - 33.08)	11.76 (10.37 - 13.48)	0.004 (-0.01 - 0.018)	74.92 (54.61 - 94.35)	103.3 (76.16 - 129.13)	210.53 (153.5 - 265.05)	106.74 (78.18 - 133.81)	0.144 (0.097 - 0.19)
Antigua and Barbuda	0.08 (0.07 - 0.09)	12.26 (10.74 - 14.12)	0.12 (0.1 - 0.14)	12.26 (10.73 - 14.08)	-0.006 (-0.018 - 0.005)	0.7 (0.52 - 0.89)	125.62 (93.28 - 157.95)	1.32 (0.97 - 1.65)	126.93 (93.15 - 159.73)	0.035 (0.025 - 0.045)
Argentina	34.07 (29.84 - 38.68)	10.66 (9.32 - 12.12)	48.13 (42.27 - 54.86)	10.57 (9.26 - 12.05)	-0.014 (-0.022 - -0.005)	373.89 (278.48 - 462.17)	116.53 (86.68 - 144.27)	551.88 (413.34 - 688.41)	116.5 (87.29 - 145.47)	0.019 (0.012 - 0.026)
Armenia	3.88 (3.36 - 4.52)	10.78 (9.36 - 12.43)	3.43 (3 - 3.98)	10.84 (9.49 - 12.55)	0.032 (0.019 - 0.046)	39.62 (29.32 - 50.11)	117.59 (87.31 - 148.13)	42.81 (31.81 - 53.77)	119.86 (89.14 - 151.94)	0.106 (0.073 - 0.139)
Australia	30.25 (26.66 -	17.13 (15.05 -	38.49 (33.83 -	17 (14.8 -	-0.019 (-0.027	384.06 (287.69	207.32 (154.52	574.2 (428.82 -	207.27 (154.32	0.008 (-0.002

	33.93)	19.22)	43.12)	19.14)	- -0.01)	- 474.89)	- 256.57)	716.84)	- 259.49)	- 0.017)
Austria	11.39 (10.02 - 13.09)	13.69 (12 - 15.7)	11.77 (10.42 - 13.38)	13.67 (12.01 - 15.7)	-0.007 (-0.01 - -0.005)	136.59 (103.16 - 167.94)	150.12 (112.93 - 185.15)	165.9 (125.29 - 204.9)	150.13 (112.51 - 187.05)	-0.007 (-0.012 - -0.001)
Azerbaijan	8.43 (7.28 - 10.04)	10.79 (9.42 - 12.56)	12.46 (10.84 - 14.56)	10.89 (9.5 - 12.61)	0.047 (0.022 - 0.072)	78.57 (58.13 - 99.85)	118.48 (87.97 - 149.16)	141.48 (104.35 - 179.41)	121.55 (89.54 - 153.59)	0.129 (0.066 - 0.192)
Bahrain	0.73 (0.63 - 0.87)	12.14 (10.65 - 13.98)	2.08 (1.8 - 2.41)	12.17 (10.6 - 14.14)	0.02 (0.016 - 0.023)	6.3 (4.62 - 7.99)	123.61 (91.24 - 155.44)	24.69 (18.17 - 31.32)	125.31 (93.21 - 157.04)	0.068 (0.061 - 0.076)
Bangladesh	147.14 (126.38 - 170.27)	14.38 (12.59 - 16.44)	238.38 (208.2 - 275.84)	14.18 (12.45 - 16.34)	-0.051 (-0.053 - -0.049)	1245.33 (914.97 - 1578.65)	157.84 (116.4 - 198.11)	2439.97 (1836.88 - 3064.75)	157.89 (118.74 - 197.47)	-0.017 (-0.03 - -0.004)
Barbados	0.38 (0.34 - 0.44)	14.14 (12.64 - 15.96)	0.42 (0.37 - 0.47)	14.05 (12.52 - 15.89)	-0.015 (-0.018 - -0.011)	3.64 (2.7 - 4.53)	140.68 (104.47 - 174.76)	5 (3.63 - 6.22)	140.19 (101.37 - 174.55)	0.001 (-0.008 - 0.009)
Belarus	10.8 (9.47 - 12.5)	10.24 (8.92 - 11.8)	9.55 (8.4 - 10.91)	10.34 (9.04 - 11.92)	0.035 (0.019 - 0.052)	132.59 (98.04 - 164.77)	113.82 (84.02 - 142.46)	138.7 (104.75 - 172.53)	116.55 (87.6 - 146.34)	0.109 (0.077 - 0.14)
Belgium	14.12 (12.42 - 16.26)	13.64 (11.93 - 15.76)	14.55 (12.74 - 16.39)	13.63 (11.86 - 15.59)	-0.017 (-0.021 - -0.012)	174.58 (130.98 - 216.28)	148.61 (110.81 - 185.74)	202.35 (151.98 - 249.69)	148.48 (110.64 - 185.02)	-0.018 (-0.025 - -0.011)
Belize	0.2 (0.18 - 0.23)	11.97 (10.6 - 13.67)	0.5 (0.44 - 0.58)	11.95 (10.53 - 13.76)	-0.022 (-0.029 - -0.015)	1.55 (1.15 - 1.97)	118.71 (88.28 - 149.91)	4.43 (3.27 - 5.57)	120.19 (88.98 - 150.53)	0.02 (0.01 - 0.03)
Benin	4.65 (4.07 - 5.4)	12.03 (10.61 - 13.68)	12.44 (10.83 - 14.37)	12.05 (10.61 - 13.77)	0.008 (0.004 - 0.011)	32.39 (23.79 - 41.42)	103.13 (75.9 - 130.18)	87.74 (64.11 - 110.68)	106.24 (78.4 - 132.46)	0.114 (0.106 - 0.122)
Bermuda	0.09 (0.08 - 0.1)	12.62 (11.02 - 14.63)	0.08 (0.07 - 0.09)	12.56 (10.95 - 14.49)	-0.003 (-0.008 - 0.002)	0.94 (0.7 - 1.19)	133.68 (99.73 - 167.95)	1.14 (0.84 - 1.42)	134.87 (99.47 - 169.7)	0.061 (0.051 - 0.071)
Bhutan	0.81 (0.69 - 0.94)	14.47 (12.62 - 16.59)	1.63 (1.41 - 1.91)	14.54 (12.72 - 16.78)	0.035 (0.023 - 0.047)	6.76 (4.97 - 8.51)	159.63 (118.69 - 199.48)	16.46 (12.04 - 21.12)	165.41 (121.98 - 207.65)	0.146 (0.137 - 0.155)
Bolivia	7.01 (6.14 - 8.2)	12.04 (10.61 - 13.84)	14.2 (12.47 - 16.42)	12.09 (10.65 - 13.84)	0.006 (-0.002 - 0.014)	56.81 (42.05 - 71.35)	116.86 (86.86 - 145.59)	128.6 (95.71 - 161.3)	120.23 (89.59 - 150.28)	0.087 (0.078 - 0.096)

Bosnia and Herzegovina	5.45 (4.74 - 6.31)	11.04 (9.6 - 12.79)	3.66 (3.23 - 4.17)	11.21 (9.78 - 13.01)	0.07 (0.062 - 0.078)	57.04 (41.94 - 71.48)	116.11 (85.52 - 145.86)	50.66 (37.83 - 63.37)	120.86 (89.76 - 152.77)	0.191 (0.163 - 0.218)
Botswana	1.36 (1.18 - 1.57)	11.71 (10.29 - 13.3)	2.96 (2.58 - 3.45)	11.87 (10.49 - 13.6)	0.046 (0.041 - 0.05)	9.67 (7.2 - 12.3)	104.88 (78.58 - 131.96)	24.47 (18.2 - 30.88)	109.29 (81.59 - 136.78)	0.154 (0.134 - 0.175)
Brazil	188.45 (164.26 - 218.24)	12.44 (10.95 - 14.21)	285.92 (252.59 - 327.84)	12.45 (10.97 - 14.29)	0 (-0.006 - 0.005)	1604.59 (1205.26 - 2000.25)	123.55 (92.57 - 152.59)	2995.27 (2235.89 - 3708.4)	125.53 (93.71 - 155.01)	0.053 (0.039 - 0.066)
Brunei	0.42 (0.36 - 0.49)	14.26 (12.54 - 16.24)	0.72 (0.63 - 0.83)	14.09 (12.37 - 16.17)	-0.042 (-0.054 - -0.03)	3.76 (2.74 - 4.77)	162.4 (119.26 - 202.07)	7.93 (5.88 - 9.95)	159.82 (119.42 - 199.23)	-0.044 (-0.053 - -0.036)
Bulgaria	9.72 (8.51 - 11.18)	11.26 (9.74 - 13.11)	7.16 (6.25 - 8.22)	11.35 (9.79 - 13.2)	0.033 (0.021 - 0.045)	123.31 (92.28 - 154.22)	120.27 (89.41 - 151.86)	107.52 (80.21 - 135.95)	122.43 (90.41 - 155.7)	0.083 (0.063 - 0.102)
Burkina Faso	8.85 (7.7 - 10.15)	11.92 (10.46 - 13.53)	21.68 (19 - 24.78)	11.94 (10.54 - 13.47)	0.004 (0 - 0.008)	61.41 (45.7 - 77.42)	98.12 (73.63 - 122.34)	154.6 (113.57 - 196.11)	103.66 (76.69 - 130.01)	0.202 (0.191 - 0.214)
Burundi	5.39 (4.73 - 6.25)	11.65 (10.35 - 13.26)	11.1 (9.7 - 12.73)	11.63 (10.3 - 13.13)	-0.013 (-0.022 - -0.003)	36.92 (27.28 - 47.04)	97.27 (72.66 - 122.43)	75.3 (55.37 - 95.86)	95.93 (70.73 - 120.85)	-0.08 (-0.12 - -0.04)
Cambodia	12.45 (10.76 - 14.43)	13.57 (11.95 - 15.49)	24.05 (20.92 - 27.68)	14.01 (12.3 - 15.95)	0.129 (0.122 - 0.135)	89.75 (66.13 - 112.81)	119.48 (88.87 - 148.27)	204.31 (150.58 - 255.3)	130.53 (97.03 - 163.25)	0.352 (0.332 - 0.371)
Cameroon	10.53 (9.2 - 12.16)	12.01 (10.6 - 13.67)	30.75 (26.9 - 35.33)	12.01 (10.59 - 13.58)	-0.005 (-0.012 - 0.002)	75.13 (54.97 - 94.39)	104.26 (77.08 - 129.73)	218.92 (161.08 - 274.67)	105.8 (78.87 - 132.31)	0.052 (0.03 - 0.075)
Canada	37.25 (35.63 - 38.68)	13.02 (12.49 - 13.51)	42.12 (40.02 - 44.28)	13.11 (12.58 - 13.67)	0.008 (-0.001 - 0.016)	571.27 (433.04 - 675.73)	182.96 (138.57 - 216.47)	790.81 (606.08 - 939.91)	183.1 (139.74 - 217.59)	0.004 (0 - 0.007)
Cape Verde	0.36 (0.31 - 0.42)	12.16 (10.71 - 13.76)	0.73 (0.64 - 0.85)	12.25 (10.81 - 14.01)	0.022 (0.016 - 0.029)	2.66 (1.95 - 3.35)	111.8 (81.75 - 139.35)	6.29 (4.65 - 7.95)	116.09 (85.46 - 145.93)	0.154 (0.142 - 0.165)
Central African Republic	2.75 (2.39 - 3.16)	11.48 (10.12 - 13.02)	5 (4.37 - 5.71)	11.4 (10.08 - 12.88)	-0.033 (-0.037 - -0.028)	18.32 (13.57 - 23.38)	92.09 (69.22 - 116.18)	34.22 (25.12 - 43.08)	92.21 (68.2 - 114.57)	-0.009 (-0.026 - 0.008)

Chad	5.72 (5.01 - 6.55)	11.94 (10.52 - 13.53)	14.33 (12.51 - 16.57)	12.01 (10.61 - 13.62)	0.024 (0.018 - 0.03)	40.26 (29.55 - 50.71)	101.1 (74.72 - 126.59)	98.16 (72.09 - 123.78)	104.43 (77.81 - 130.23)	0.13 (0.105 - 0.155)
Chile	15.08 (13.13 - 17.39)	10.71 (9.37 - 12.2)	20.12 (17.63 - 23)	10.64 (9.32 - 12.18)	-0.004 (-0.015 - 0.006)	147.5 (108.2 - 185.68)	116.27 (86.11 - 145.4)	243.22 (180.41 - 305.33)	117.39 (86.74 - 147.5)	0.051 (0.043 - 0.059)
China	2707.99 (2421.62 - 3053.8)	20.08 (18.11 - 22.46)	2918.89 (2636.85 - 3272.64)	19.81 (17.87 - 22.14)	-0.059 (-0.067 - -0.05)	23510.53 (17641.14 - 28945.46)	198.75 (149.35 - 243.74)	36684.7 (27513.14 - 45015.38)	207.07 (154.78 - 255.31)	0.136 (0.117 - 0.155)
Colombia	40.92 (35.63 - 47.59)	12.23 (10.71 - 14.05)	64.45 (56.49 - 73.77)	12.3 (10.78 - 14.13)	0.004 (-0.002 - 0.01)	341.98 (250.24 - 438.85)	123.95 (90.95 - 156.46)	670.16 (497.28 - 840.13)	127.18 (94.16 - 159.77)	0.06 (0.045 - 0.075)
Comoros	0.47 (0.41 - 0.54)	11.92 (10.53 - 13.6)	0.84 (0.73 - 0.96)	11.88 (10.46 - 13.51)	-0.017 (-0.024 - -0.01)	3.35 (2.47 - 4.22)	105.45 (78.57 - 132.11)	6.71 (5 - 8.38)	105.56 (78.7 - 131.79)	0.006 (-0.003 - 0.015)
Congo	2.49 (2.17 - 2.88)	11.69 (10.34 - 13.34)	5.68 (4.99 - 6.51)	11.72 (10.39 - 13.33)	0.006 (-0.002 - 0.014)	17.39 (12.86 - 21.92)	102.11 (75.98 - 127.1)	45.14 (33.25 - 57.03)	104.85 (77.78 - 131.57)	0.092 (0.065 - 0.12)
Costa Rica	3.82 (3.31 - 4.48)	12.41 (10.92 - 14.24)	6.32 (5.49 - 7.33)	12.46 (10.86 - 14.39)	0.003 (-0.002 - 0.008)	32.7 (23.91 - 41.54)	127.8 (94.24 - 159.68)	66.7 (48.77 - 83.97)	130.26 (95.25 - 164.09)	0.05 (0.043 - 0.057)
Cote d'Ivoire	12.79 (11.06 - 14.95)	11.97 (10.55 - 13.62)	28.47 (24.91 - 32.78)	11.99 (10.64 - 13.58)	-0.001 (-0.008 - 0.007)	87.8 (64.98 - 111.37)	103.89 (78.33 - 128.7)	210.16 (155.96 - 269.09)	106.04 (79.78 - 134.23)	0.035 (0.018 - 0.052)
Croatia	5.6 (4.91 - 6.49)	11.28 (9.82 - 13.14)	4.43 (3.9 - 5.02)	11.33 (9.9 - 13.08)	0.038 (0.027 - 0.049)	70.26 (52.13 - 87.82)	122.86 (91.37 - 154.88)	64.65 (48.56 - 80.25)	124.06 (93.14 - 155.68)	0.072 (0.058 - 0.086)
Cuba	15.01 (13.05 - 17.46)	12.23 (10.72 - 14.07)	13.92 (12.3 - 15.85)	12.17 (10.74 - 14.07)	-0.007 (-0.02 - 0.006)	139.15 (103.57 - 175.79)	124.45 (92.75 - 156.15)	175.87 (129.01 - 217.82)	125.13 (91.83 - 156.3)	0.053 (0.028 - 0.078)
Cyprus	1.1 (0.97 - 1.27)	13.64 (11.96 - 15.7)	1.89 (1.66 - 2.17)	13.61 (11.94 - 15.63)	-0.006 (-0.012 - 0)	12.2 (9.12 - 15.22)	148.85 (111.24 - 185.66)	23.7 (17.6 - 29.5)	149.19 (110.42 - 185.99)	0.012 (0.006 - 0.019)
Czech Republic	11.09 (9.68 - 12.7)	11.05 (9.58 - 12.84)	10.65 (9.3 - 12.24)	11.09 (9.55 - 12.92)	0.016 (0.013 - 0.019)	142.81 (105.06 - 178.57)	123.33 (90.25 - 154.58)	164.89 (124.93 - 204.8)	124.85 (93.82 - 156.86)	0.038 (0.033 - 0.042)

Democratic Republic of the Congo	37.71 (32.8 - 43.77)	11.63 (10.28 - 13.29)	83.86 (73.34 - 95.81)	11.58 (10.24 - 13.12)	-0.019 (-0.03 - -0.008)	259.52 (191.8 - 331.06)	97.97 (72.25 - 123.5)	585.48 (432.97 - 745.8)	97.05 (72.12 - 122.16)	0.001 (-0.054 - 0.057)
Denmark	7.29 (6.53 - 8.12)	13.41 (12.02 - 15.03)	7.95 (7.02 - 8.94)	14.38 (12.53 - 16.29)	0.355 (0.265 - 0.444)	84.43 (63.95 - 103.72)	138.41 (104.74 - 170.59)	101.55 (75.82 - 124.98)	148.45 (110.53 - 183.97)	0.352 (0.261 - 0.444)
Djibouti	0.56 (0.48 - 0.65)	11.93 (10.5 - 13.59)	1.39 (1.22 - 1.61)	11.93 (10.58 - 13.56)	-0.005 (-0.012 - 0.002)	3.82 (2.81 - 4.84)	108.98 (80.92 - 136.83)	11.43 (8.46 - 14.45)	108.6 (80.77 - 136.56)	-0.022 (-0.048 - 0.005)
Dominica	0.09 (0.07 - 0.1)	12.1 (10.57 - 13.99)	0.08 (0.07 - 0.1)	12.1 (10.59 - 13.93)	-0.012 (-0.019 - -0.005)	0.77 (0.57 - 0.97)	121.36 (89.93 - 153.3)	0.93 (0.7 - 1.17)	123.04 (91.85 - 154.5)	0.049 (0.041 - 0.056)
Dominican Republic	8.59 (7.45 - 10.08)	12 (10.51 - 13.94)	13.6 (11.91 - 15.67)	12.08 (10.62 - 13.86)	0.033 (0.026 - 0.04)	68.37 (50.55 - 87.03)	120.06 (89.01 - 151.43)	130.55 (96.83 - 164.89)	123.07 (91.18 - 154.69)	0.1 (0.091 - 0.109)
Ecuador	11.92 (10.33 - 13.84)	12.25 (10.75 - 14.04)	21.29 (18.67 - 24.49)	12.26 (10.75 - 14.05)	-0.002 (-0.014 - 0.01)	97.92 (73.29 - 123.1)	123.33 (92.35 - 154.59)	204.86 (151.06 - 258.9)	124.84 (91.82 - 156.49)	0.035 (0.018 - 0.051)
Egypt	61.35 (53.58 - 71.24)	11.56 (10.15 - 13.31)	115.52 (101.44 - 133.88)	11.59 (10.22 - 13.31)	0.023 (0.017 - 0.028)	498.38 (365.74 - 625.53)	110.96 (81.47 - 138.28)	1027.55 (756.36 - 1293.79)	114.3 (84.43 - 142.93)	0.108 (0.102 - 0.114)
El Salvador	5.85 (5.08 - 6.79)	11.97 (10.52 - 13.76)	7.77 (6.81 - 8.98)	12.04 (10.66 - 13.8)	0.011 (0.006 - 0.016)	46.93 (34.36 - 59.06)	117.68 (86.34 - 147.31)	72.22 (53.91 - 91.12)	121.07 (89.98 - 152.27)	0.1 (0.092 - 0.108)
Equatorial Guinea	0.4 (0.35 - 0.46)	11.55 (10.25 - 13.14)	1.57 (1.36 - 1.83)	12 (10.59 - 13.85)	0.178 (0.156 - 0.2)	2.8 (2.09 - 3.55)	95.02 (70.64 - 119.65)	10.99 (7.93 - 13.92)	113.76 (83.76 - 141.95)	0.83 (0.71 - 0.95)
Eritrea	2.84 (2.48 - 3.27)	11.64 (10.34 - 13.21)	6.61 (5.79 - 7.64)	11.65 (10.32 - 13.23)	-0.004 (-0.007 - -0.001)	18 (13.19 - 22.75)	93.22 (68.67 - 117.38)	44.94 (33.05 - 56.86)	97.62 (71.54 - 122.39)	0.136 (0.101 - 0.171)
Estonia	1.6 (1.41 - 1.85)	10.32 (9.03 - 12.05)	1.27 (1.12 - 1.47)	10.46 (9.06 - 12.11)	0.051 (0.042 - 0.061)	20.5 (15.15 - 25.77)	115.95 (85.25 - 146.04)	19.15 (14.21 - 23.84)	119.53 (89.35 - 148.92)	0.138 (0.12 - 0.155)
Ethiopia	49.93 (43.8 - 57.18)	11.95 (10.6 - 13.57)	110.64 (96.63 - 127.39)	11.98 (10.62 - 13.58)	-0.001 (-0.01 - 0.009)	332.71 (247.25 - 415.87)	96.67 (72.48 - 120.31)	751.53 (561.78 - 944.08)	102.98 (76.77 - 126.94)	0.253 (0.203 - 0.303)

Federated States of Micronesia	0.14 (0.12 - 0.16)	14.68 (12.98 - 16.75)	0.16 (0.14 - 0.18)	14.69 (12.93 - 16.77)	0.017 (0.011 - 0.023)	1.02 (0.75 - 1.29)	134.94 (99.96 - 168.7)	1.31 (0.96 - 1.64)	135.13 (99.08 - 168.68)	0.015 (0.007 - 0.023)
Fiji	1.17 (1.02 - 1.35)	15.1 (13.29 - 17.26)	1.42 (1.24 - 1.62)	15.17 (13.29 - 17.31)	0.034 (0.029 - 0.039)	9.5 (6.91 - 11.96)	144.06 (106.91 - 179.19)	13.51 (9.99 - 16.92)	146.05 (108.08 - 182.48)	0.065 (0.051 - 0.079)
Finland	6.95 (6.14 - 7.81)	13.39 (11.82 - 15.15)	6.46 (5.66 - 7.31)	12.59 (10.98 - 14.4)	-0.137 (-0.189 - -0.086)	94.13 (71.08 - 116.18)	156.74 (118.28 - 193.83)	100.49 (76.17 - 124.31)	149.39 (112.5 - 187.18)	-0.089 (-0.132 - -0.047)
France	80.65 (71.11 - 92.46)	13.59 (11.92 - 15.63)	81.23 (71.78 - 91.51)	13.53 (11.84 - 15.47)	-0.022 (-0.025 - -0.019)	970.15 (710.46 - 1201.72)	148.95 (109.01 - 185.81)	1138.58 (854.14 - 1413.28)	148.28 (110.5 - 184.97)	-0.022 (-0.027 - -0.017)
Gabon	1.06 (0.92 - 1.22)	11.93 (10.5 - 13.49)	2.09 (1.82 - 2.4)	11.93 (10.5 - 13.59)	0.007 (0.002 - 0.012)	8.21 (6 - 10.38)	110.52 (81.06 - 137.47)	17.14 (12.75 - 21.5)	111.83 (83.72 - 139.54)	0.053 (0.044 - 0.061)
Georgia	6.08 (5.33 - 7.01)	10.78 (9.47 - 12.4)	3.82 (3.37 - 4.37)	10.77 (9.43 - 12.43)	-0.001 (-0.019 - 0.018)	70.68 (52.72 - 88.06)	119.5 (88.74 - 149.64)	51.53 (38.02 - 64.52)	119.04 (87.31 - 149.28)	0.01 (-0.04 - 0.059)
Germany	118.53 (104.02 - 136.58)	13.87 (12.12 - 15.92)	109.54 (96.74 - 124.21)	13.89 (12.11 - 16.01)	-0.041 (-0.056 - -0.026)	1402.99 (1043.85 - 1717.81)	143.67 (106.59 - 176.74)	1507 (1130.56 - 1875.95)	144.48 (107.61 - 181.18)	-0.047 (-0.069 - -0.025)
Ghana	15.81 (13.83 - 18.32)	12.01 (10.65 - 13.69)	36.62 (32 - 42.09)	12.08 (10.69 - 13.74)	0.017 (0.012 - 0.022)	113.7 (83.74 - 143.73)	104.99 (78.16 - 132.07)	280.33 (206.65 - 353.4)	109.24 (81.7 - 136.09)	0.121 (0.104 - 0.137)
Greece	14.44 (12.73 - 16.45)	13.65 (11.98 - 15.68)	12.94 (11.42 - 14.54)	13.58 (11.92 - 15.51)	-0.009 (-0.017 - 0)	178.93 (134.83 - 222.06)	149.26 (111.73 - 185.31)	192.87 (143.93 - 237.42)	148.08 (110.99 - 184.75)	-0.006 (-0.016 - 0.004)
Greenland	0.1 (0.08 - 0.11)	13.85 (12.2 - 15.67)	0.08 (0.07 - 0.09)	13.7 (12.04 - 15.65)	-0.034 (-0.054 - -0.014)	1.14 (0.86 - 1.42)	187.52 (141.13 - 232.06)	1.24 (0.93 - 1.54)	187.75 (141.83 - 234.52)	0.037 (0.013 - 0.06)
Grenada	0.1 (0.08 - 0.11)	12.02 (10.6 - 13.72)	0.14 (0.12 - 0.16)	12.11 (10.62 - 13.95)	0.02 (0.015 - 0.025)	0.82 (0.62 - 1.03)	120.06 (90.25 - 149.42)	1.45 (1.08 - 1.83)	123.35 (91.49 - 155.55)	0.102 (0.096 - 0.108)
Guam	0.24 (0.21 - 0.29)	15.85 (13.93 - 18.5)	0.27 (0.23 - 0.3)	15.77 (13.8 - 18.12)	-0.006 (-0.013 - 0.001)	2.16 (1.59 - 2.76)	161.18 (119.66 - 204.19)	2.77 (2.04 - 3.45)	159.24 (117.07 - 199.12)	-0.029 (-0.036 - -0.022)

Guatemala	8.14 (7.07 - 9.41)	11.97 (10.53 - 13.6)	21.33 (18.41 - 24.93)	11.96 (10.47 - 13.74)	-0.004 (-0.006 - -0.002)	65.17 (48.15 - 83.21)	116.45 (86.72 - 147.5)	175.66 (129.09 - 221.88)	118.74 (88.22 - 148.2)	0.059 (0.053 - 0.064)
Guinea	6.07 (5.34 - 6.94)	12.01 (10.63 - 13.67)	12.38 (10.8 - 14.24)	12 (10.62 - 13.55)	-0.002 (-0.007 - 0.002)	45.84 (34.34 - 57.32)	103.69 (77.16 - 129.72)	87.59 (64.9 - 111.02)	103.94 (77.95 - 130.75)	-0.005 (-0.014 - 0.004)
Guinea-Bissau	0.98 (0.85 - 1.13)	11.92 (10.51 - 13.51)	2.04 (1.77 - 2.37)	11.9 (10.48 - 13.57)	-0.015 (-0.021 - -0.009)	6.72 (5 - 8.55)	100.47 (74.72 - 125.54)	13.87 (10.28 - 17.59)	101.22 (74.95 - 127.2)	-0.006 (-0.02 - 0.007)
Guyana	0.92 (0.8 - 1.08)	11.74 (10.34 - 13.47)	0.91 (0.8 - 1.04)	11.74 (10.29 - 13.45)	-0.01 (-0.016 - -0.004)	7.01 (5.17 - 8.87)	111.66 (83.14 - 139.59)	8.44 (6.25 - 10.56)	114.83 (85.39 - 143.81)	0.088 (0.079 - 0.097)
Haiti	6.58 (5.76 - 7.62)	11.54 (10.18 - 13.19)	13.99 (12.24 - 16.37)	11.45 (10.12 - 13.15)	-0.032 (-0.039 - -0.025)	51.66 (38.2 - 65.44)	106.34 (79.25 - 133.59)	114.08 (85 - 143.75)	107 (79.96 - 133.54)	0.035 (0.024 - 0.045)
Honduras	4.73 (4.11 - 5.48)	11.92 (10.51 - 13.76)	11.43 (9.93 - 13.32)	11.91 (10.42 - 13.67)	-0.009 (-0.01 - -0.007)	36.98 (27.35 - 46.95)	116.64 (87.17 - 147.01)	95.01 (70.24 - 120.59)	117.61 (87.28 - 147.75)	0.026 (0.021 - 0.031)
Hungary	11.31 (10.01 - 12.85)	11.16 (9.75 - 12.9)	10.14 (8.92 - 11.54)	11.26 (9.85 - 13.01)	0.038 (0.034 - 0.042)	143.49 (106.24 - 176.72)	119.72 (88.48 - 149.36)	148.12 (110.74 - 184.36)	122.75 (90.26 - 154.98)	0.101 (0.096 - 0.106)
Iceland	0.37 (0.32 - 0.42)	13.79 (12.1 - 15.77)	0.46 (0.41 - 0.53)	13.74 (12.09 - 15.84)	-0.014 (-0.019 - -0.009)	4.01 (3 - 4.98)	151.92 (113.09 - 188.44)	5.92 (4.39 - 7.35)	151.71 (112.5 - 189.65)	-0.001 (-0.008 - 0.006)
India	1306.27 (1135.75 - 1503.45)	15.04 (13.17 - 17.24)	2143.68 (1869.39 - 2453.72)	14.47 (12.68 - 16.55)	-0.091 (-0.124 - -0.057)	12138.98 (9105.33 - 14998.24)	163.28 (122.88 - 201.78)	22102.81 (16650.77 - 27381.98)	160.59 (120.92 - 198.33)	-0.007 (-0.034 - 0.021)
Indonesia	265.6 (230.74 - 306.84)	14.13 (12.44 - 16.08)	399.19 (352.11 - 452.75)	14.55 (12.83 - 16.58)	0.095 (0.088 - 0.102)	2152 (1595.25 - 2693.46)	134.64 (99.65 - 167.25)	3967.31 (2937.42 - 4921.47)	143.64 (106.2 - 177.7)	0.203 (0.187 - 0.218)
Iran	63.96 (55.29 - 74.51)	11.96 (10.48 - 13.69)	111.58 (98.05 - 129.95)	11.95 (10.49 - 13.71)	0.025 (0.016 - 0.033)	495.91 (369.01 - 620)	118.26 (88.18 - 146.82)	1139.36 (846.5 - 1423.98)	120.49 (89.9 - 149.87)	0.099 (0.088 - 0.11)
Iraq	17.8 (15.51 - 20.69)	11.64 (10.18 - 13.41)	50.63 (43.77 - 58.68)	11.63 (10.19 - 13.34)	0.019 (0.007 - 0.03)	135.76 (100.06 - 170.82)	111.88 (82.79 - 140.52)	419.9 (305.73 - 525.85)	114.28 (84.15 - 142.01)	0.126 (0.096 - 0.156)

Ireland	5.31 (4.6 - 6.07)	14.99 (12.95 - 17.22)	7 (6.12 - 8.07)	15.65 (13.58 - 18.19)	0.196 (0.166 - 0.226)	64.96 (47.88 - 80.98)	181.76 (133.47 - 227.58)	107.37 (79.85 - 133.7)	190.84 (142.25 - 237.76)	0.209 (0.189 - 0.23)
Israel	6.82 (5.89 - 7.83)	14.2 (12.29 - 16.33)	11.9 (10.42 - 13.61)	13.99 (12.18 - 16.1)	-0.01 (-0.024 - 0.005)	72.73 (54.26 - 90.42)	156.07 (116.06 - 194.11)	140.22 (104.75 - 175.43)	154.01 (115.15 - 193.26)	0.013 (-0.005 - 0.03)
Italy	79.36 (76.98 - 82.12)	13.25 (12.87 - 13.69)	71.81 (68.44 - 75.38)	13.1 (12.47 - 13.65)	-0.024 (-0.035 - -0.013)	940.87 (710.95 - 1135.33)	140.03 (105.73 - 169.02)	1061.1 (800.95 - 1287.74)	139.55 (105 - 168.74)	-0.007 (-0.012 - -0.002)
Jamaica	2.83 (2.45 - 3.31)	12.14 (10.58 - 14.01)	3.72 (3.25 - 4.28)	12.08 (10.62 - 13.84)	-0.026 (-0.032 - -0.021)	23.93 (17.71 - 30.2)	123.27 (91.36 - 154.98)	36.56 (27.21 - 45.68)	123.02 (91.4 - 153.81)	-0.018 (-0.025 - -0.011)
Japan	189.34 (167.08 - 213.94)	14.93 (13.07 - 17.19)	163.27 (144.72 - 182.89)	15.2 (13.34 - 17.45)	0.187 (0.13 - 0.245)	2537.41 (1885.47 - 3130.1)	170.41 (126.79 - 211.05)	2662.3 (1988.72 - 3264.94)	171.19 (127.4 - 212)	0.128 (0.075 - 0.18)
Jordan	4.22 (3.58 - 4.99)	11.76 (10.27 - 13.58)	13.3 (11.56 - 15.36)	11.75 (10.29 - 13.49)	0.005 (-0.003 - 0.012)	30.24 (22.14 - 38.38)	116.12 (85.48 - 146.41)	116.41 (86.38 - 147.2)	117.41 (87.54 - 146.71)	0.06 (0.047 - 0.073)
Kazakhstan	18.7 (16.23 - 21.72)	10.71 (9.35 - 12.31)	20.28 (17.67 - 23.8)	10.88 (9.51 - 12.61)	0.061 (0.041 - 0.081)	188.01 (139.56 - 236.61)	116.67 (86.96 - 146.52)	233.03 (172.41 - 294.29)	120.77 (89.4 - 152.15)	0.143 (0.098 - 0.187)
Kenya	23.04 (20.09 - 26.6)	11.93 (10.61 - 13.56)	55.51 (48.63 - 63.58)	11.93 (10.61 - 13.52)	-0.009 (-0.014 - -0.004)	155.75 (115.73 - 194.41)	105.6 (79.29 - 130.85)	402.9 (299.44 - 503.86)	106.11 (79.49 - 131.12)	0.013 (-0.008 - 0.034)
Kiribati	0.11 (0.09 - 0.12)	14.49 (12.82 - 16.45)	0.17 (0.15 - 0.2)	14.42 (12.7 - 16.4)	-0.003 (-0.008 - 0.002)	0.8 (0.59 - 1)	130.58 (96.96 - 161.22)	1.35 (1 - 1.7)	128.28 (95.16 - 160.38)	-0.041 (-0.05 - -0.032)
Kuwait	2.59 (2.22 - 3.08)	12.18 (10.64 - 14.02)	6.55 (5.67 - 7.69)	12.23 (10.72 - 14.14)	0.025 (0.019 - 0.032)	22.34 (16.34 - 28.4)	126.06 (93.22 - 157.88)	69.96 (50.98 - 89.62)	127.35 (94.03 - 160.39)	0.065 (0.051 - 0.079)
Kyrgyzstan	4.62 (4 - 5.4)	10.59 (9.29 - 12.23)	7.12 (6.15 - 8.3)	10.52 (9.19 - 12.14)	-0.022 (-0.034 - -0.01)	42.22 (31.27 - 52.86)	113.19 (84.05 - 140.97)	68.55 (50.82 - 87.04)	111.84 (83.25 - 140.2)	-0.039 (-0.075 - -0.002)
Laos	5.04 (4.4 - 5.81)	13.67 (12.08 - 15.55)	10.57 (9.2 - 12.19)	14.15 (12.42 - 16.07)	0.13 (0.123 - 0.136)	38.28 (28.14 - 48.44)	123.45 (91.55 - 155.21)	88.3 (65.37 - 110.41)	135.06 (100.62 - 168.39)	0.33 (0.316 - 0.344)
Latvia	2.79 (2.45 -	10.33 (9.01 -	1.84 (1.63 -	10.38 (9.09 -	0.035 (0.018 -	35.74 (26.87 -	115.83 (86.55 -	28.14 (21.01 -	117.65 (87.79 -	0.097 (0.066 -

	3.22)	11.99)	2.11)	12.05)	0.052)	44.29)	144.9)	35.15)	147.72)	0.129)
Lebanon	4.5 (3.89 - 5.2)	11.81 (10.34 - 13.7)	10.98 (9.5 - 12.81)	11.8 (10.29 - 13.58)	0.006 (-0.002 - 0.014)	36.74 (27.22 - 46.49)	117.05 (86.96 - 147.64)	98.85 (72.43 - 125.05)	118.31 (87.74 - 148.02)	0.062 (0.048 - 0.076)
Lesotho	1.82 (1.6 - 2.08)	11.44 (10.11 - 12.98)	2.3 (2.01 - 2.66)	11.48 (10.11 - 12.98)	0.013 (0.008 - 0.018)	12.8 (9.47 - 16.09)	94.76 (69.9 - 119.11)	16.95 (12.6 - 21.26)	96.85 (72.34 - 120.11)	0.074 (0.054 - 0.093)
Liberia	1.86 (1.65 - 2.12)	11.98 (10.62 - 13.65)	5.2 (4.53 - 5.97)	11.92 (10.53 - 13.56)	-0.01 (-0.018 - -0.003)	14.36 (10.52 - 18.27)	101.07 (74.74 - 127.9)	36.56 (27.11 - 46.07)	99 (73.69 - 123.11)	0.01 (-0.065 - 0.084)
Libya	4.73 (4.08 - 5.51)	11.91 (10.44 - 13.72)	8.95 (7.84 - 10.36)	11.69 (10.27 - 13.47)	-0.033 (-0.046 - -0.021)	36.59 (27.07 - 46.1)	120.11 (89.15 - 150.46)	85.32 (63.27 - 107.16)	115.28 (86.8 - 143.75)	-0.084 (-0.114 - -0.054)
Lithuania	3.98 (3.49 - 4.65)	10.33 (9.05 - 12.04)	2.77 (2.44 - 3.17)	10.44 (9.15 - 12.06)	0.042 (0.027 - 0.057)	48.04 (35.73 - 59.82)	116.02 (86.28 - 144.87)	41.17 (30.75 - 50.99)	118.66 (88.02 - 147.96)	0.111 (0.082 - 0.139)
Luxembourg	0.58 (0.51 - 0.66)	13.81 (12.06 - 15.8)	0.84 (0.74 - 0.96)	13.88 (12.07 - 15.95)	0.018 (0.013 - 0.024)	7.11 (5.36 - 8.8)	151.06 (113.39 - 187.22)	11.4 (8.48 - 14.17)	153.36 (114.4 - 191.43)	0.051 (0.044 - 0.057)
Macedonia	2.37 (2.06 - 2.75)	11.19 (9.73 - 13.01)	2.52 (2.22 - 2.9)	11.24 (9.81 - 13.08)	0.01 (0.007 - 0.013)	25.62 (18.92 - 32.39)	121.17 (89.35 - 153.21)	32.87 (24.57 - 41.11)	122.54 (91.5 - 154.55)	0.044 (0.033 - 0.054)
Madagascar	12.1 (10.57 - 13.99)	11.85 (10.5 - 13.54)	27.84 (24.33 - 32.03)	11.81 (10.49 - 13.38)	-0.011 (-0.019 - -0.003)	85.42 (63.76 - 108.08)	103.37 (77.59 - 129.79)	199.26 (147.16 - 253.1)	102.93 (76.99 - 129.51)	0.002 (-0.016 - 0.019)
Malawi	9.48 (8.32 - 10.89)	11.6 (10.31 - 13.11)	17.82 (15.55 - 20.55)	11.58 (10.27 - 13.18)	-0.004 (-0.009 - 0.001)	62.88 (46.44 - 79.88)	94.81 (70.64 - 119.59)	119.31 (87.56 - 149.8)	96.06 (71.06 - 119.42)	0.081 (0.041 - 0.12)
Malaysia	25.86 (22.46 - 29.91)	14.51 (12.81 - 16.59)	51.56 (44.99 - 59.84)	15.02 (13.15 - 17.27)	0.126 (0.119 - 0.133)	219.21 (160.17 - 276.58)	143.92 (105.91 - 180.22)	498.87 (363.24 - 625.66)	153.24 (111.91 - 191.81)	0.221 (0.211 - 0.231)
Maldives	0.28 (0.24 - 0.33)	14.35 (12.59 - 16.43)	0.86 (0.75 - 1.02)	15.07 (13.22 - 17.29)	0.186 (0.171 - 0.201)	2.1 (1.54 - 2.66)	138.53 (102.91 - 173.05)	8.27 (6.09 - 10.5)	152.11 (113.03 - 191.99)	0.357 (0.347 - 0.368)
Mali	8.4 (7.36 - 9.68)	11.97 (10.54 - 13.72)	20.22 (17.56 - 23.31)	12.01 (10.6 - 13.66)	0.011 (0.007 - 0.015)	60.51 (44.79 - 76.38)	100.53 (73.94 - 126.43)	140.42 (103.13 - 177.78)	104.54 (77.3 - 130.85)	0.15 (0.141 - 0.158)
Malta	0.52 (0.45 -	13.66 (11.99 -	0.58 (0.52 -	13.72 (12.04 -	0.015 (0.012 -	6.2 (4.66 -	149.31 (111.79 -	8.16 (6.07 -	150.53 (111.34 -	0.026 (0.022 -

	0.59)	15.62)	0.66)	15.79)	0.018)	7.73)	186.4)	10.05)	186.61)	0.03)
Marshall Islands	0.06 (0.05 - 0.07)	14.63 (12.97 - 16.65)	0.08 (0.07 - 0.1)	14.65 (12.85 - 16.72)	0.013 (0.009 - 0.017)	0.41 (0.3 - 0.52)	134.58 (100.17 - 169.39)	0.72 (0.53 - 0.9)	135.15 (99.35 - 168.33)	0.02 (0.009 - 0.03)
Mauritania	2.15 (1.87 - 2.49)	12.14 (10.65 - 13.85)	4.31 (3.73 - 4.98)	12.16 (10.58 - 13.8)	0.004 (-0.002 - 0.01)	16.03 (11.59 - 20.14)	109.2 (80.09 - 136.05)	32.97 (24.66 - 41.82)	111.46 (83.01 - 140.81)	0.078 (0.065 - 0.092)
Mauritius	1.74 (1.51 - 2.03)	14.39 (12.63 - 16.46)	2 (1.77 - 2.28)	14.86 (13.09 - 17.08)	0.113 (0.105 - 0.12)	15.19 (11.2 - 19.17)	139.95 (103.23 - 175.48)	22.45 (16.88 - 27.86)	147.96 (111.38 - 184.3)	0.183 (0.17 - 0.196)
Mexico	103.82 (90.27 - 120.73)	12.5 (11.04 - 14.33)	165.1 (145.14 - 189.34)	12.51 (11.01 - 14.35)	-0.001 (-0.003 - 0)	843.81 (625.59 - 1054.45)	127.43 (94.58 - 158.09)	1665.5 (1235.02 - 2066.79)	128.33 (95.18 - 159.09)	0.017 (0.012 - 0.022)
Moldova	4.55 (4.01 - 5.23)	10.18 (8.91 - 11.7)	3.9 (3.43 - 4.46)	10.11 (8.86 - 11.59)	-0.021 (-0.032 - -0.01)	52.09 (38.91 - 64.48)	111.45 (83.31 - 137.97)	51.48 (38.03 - 64.37)	110.48 (81.67 - 138.65)	-0.019 (-0.056 - 0.017)
Mongolia	2.21 (1.9 - 2.59)	10.54 (9.21 - 12.15)	3.79 (3.3 - 4.43)	10.67 (9.33 - 12.33)	0.041 (0.031 - 0.051)	17.94 (12.94 - 22.82)	111.8 (81.5 - 140.35)	40.28 (29.68 - 50.37)	115.84 (85.47 - 144.27)	0.134 (0.106 - 0.163)
Montenegro	0.73 (0.63 - 0.85)	11.2 (9.71 - 13)	0.68 (0.59 - 0.78)	11.24 (9.72 - 13.07)	0.02 (0.011 - 0.029)	8 (5.93 - 10.06)	121.25 (89.63 - 152.33)	9.03 (6.68 - 11.32)	122.12 (89.43 - 154.63)	0.051 (0.035 - 0.066)
Morocco	28.52 (24.77 - 33.03)	11.53 (10.13 - 13.12)	43.49 (38.16 - 50.4)	11.55 (10.14 - 13.35)	0.007 (0.003 - 0.011)	226.3 (167.25 - 284.24)	110.28 (82 - 137.12)	418.67 (310.84 - 526.41)	112.58 (83.7 - 141.29)	0.071 (0.065 - 0.077)
Mozambique	13.98 (12.25 - 16.04)	11.6 (10.24 - 13.12)	30.29 (26.51 - 34.76)	11.62 (10.27 - 13.08)	0.017 (0.01 - 0.024)	92.29 (67.62 - 116.84)	91.75 (67.94 - 115.04)	198.27 (147.2 - 250.64)	96.46 (72.34 - 121.2)	0.226 (0.204 - 0.248)
Myanmar	54.8 (47.83 - 62.85)	13.59 (11.99 - 15.37)	79.32 (69.61 - 90.59)	14.08 (12.39 - 16.08)	0.154 (0.144 - 0.164)	411.21 (301.11 - 518.8)	119.39 (88.37 - 148.68)	731.27 (537.27 - 914.69)	133.35 (97.87 - 166.33)	0.459 (0.429 - 0.49)
Namibia	1.49 (1.3 - 1.73)	11.72 (10.33 - 13.36)	2.8 (2.46 - 3.22)	11.76 (10.43 - 13.33)	0.01 (0.002 - 0.019)	10.82 (8 - 13.73)	105.34 (78.23 - 131.94)	22.06 (16.23 - 27.92)	107.68 (79.62 - 134.52)	0.087 (0.054 - 0.12)
Nepal	25.48 (22.13 - 29.66)	14.23 (12.46 - 16.37)	44.11 (37.98 - 50.9)	13.96 (12.24 - 16.04)	-0.072 (-0.085 - -0.06)	226.84 (167.15 - 285.66)	152.48 (112.73 - 190.78)	418.55 (312.43 - 520.31)	152.41 (114.27 - 189.43)	-0.008 (-0.014 - -0.001)

Netherlands	33.15 (29.67 - 37.2)	20.27 (18.15 - 22.8)	31.53 (28.14 - 35.13)	19.48 (17.29 - 21.96)	-0.083 (-0.119 - -0.046)	377.95 (282.62 - 461.6)	216.97 (162.22 - 264.65)	429.8 (323.1 - 528.39)	207.99 (155.68 - 255.34)	-0.085 (-0.127 - -0.043)
New Zealand	5.95 (5.19 - 6.82)	16.6 (14.48 - 19.02)	6.69 (5.9 - 7.58)	16.43 (14.34 - 18.8)	-0.026 (-0.036 - -0.017)	70.65 (52.65 - 87.7)	193.44 (144.23 - 240.04)	96.37 (72.69 - 118.96)	191.82 (143.97 - 238.59)	-0.013 (-0.023 - -0.004)
Nicaragua	4.08 (3.52 - 4.71)	12.1 (10.62 - 13.77)	8.18 (7.17 - 9.48)	12.11 (10.67 - 13.9)	0.005 (0.001 - 0.008)	30.83 (23.01 - 39.12)	120.01 (89.73 - 150.49)	72.83 (53.79 - 91.12)	121.83 (90.55 - 151.72)	0.061 (0.054 - 0.068)
Niger	7.58 (6.64 - 8.74)	11.97 (10.59 - 13.63)	19.75 (17.29 - 22.78)	11.96 (10.6 - 13.57)	-0.007 (-0.014 - -0.001)	52.66 (38.88 - 66.93)	102.15 (75.91 - 128.83)	133.35 (97.11 - 168.54)	103.02 (75.83 - 128.61)	0.022 (0.004 - 0.041)
Nigeria	98.88 (86.65 - 113.66)	12.37 (10.9 - 13.97)	226.74 (197.79 - 261.4)	12.46 (11.02 - 14.18)	0.033 (0.023 - 0.042)	722.23 (526.45 - 902.06)	108.79 (80.07 - 135.58)	1666.89 (1226.84 - 2104.16)	113 (84.74 - 141.28)	0.198 (0.152 - 0.244)
North Korea	34.44 (30.18 - 39.58)	15.83 (13.94 - 18)	43.09 (38.16 - 48.88)	15.31 (13.54 - 17.48)	-0.133 (-0.152 - -0.114)	318.55 (236.73 - 401.38)	154.08 (113.96 - 193.51)	448.28 (334.48 - 564.34)	146.6 (108.48 - 184.69)	-0.227 (-0.272 - -0.182)
Northern Mariana Islands	0.09 (0.08 - 0.1)	15.89 (13.93 - 18.12)	0.07 (0.06 - 0.08)	15.69 (13.83 - 18.01)	-0.029 (-0.037 - -0.021)	0.78 (0.58 - 1)	162.1 (121.13 - 203.03)	0.81 (0.61 - 1.01)	157.71 (117.79 - 197.53)	-0.071 (-0.085 - -0.057)
Norway	5.15 (4.54 - 5.87)	11.77 (10.34 - 13.48)	6.12 (5.41 - 6.91)	11.73 (10.27 - 13.44)	-0.011 (-0.015 - -0.007)	58.89 (44.42 - 72.59)	122.74 (91.92 - 151.32)	77.56 (58.34 - 95.31)	122.98 (92.45 - 151.74)	0.011 (0.007 - 0.014)
Oman	2.27 (1.95 - 2.67)	11.99 (10.45 - 13.79)	7.47 (6.35 - 8.9)	12.05 (10.56 - 13.77)	0.036 (0.03 - 0.043)	18.83 (13.64 - 24.01)	120.29 (88.21 - 151.16)	68.52 (49.74 - 87.85)	123.26 (91.3 - 155.21)	0.101 (0.093 - 0.109)
Pakistan	141.9 (122.49 - 163.63)	14.49 (12.65 - 16.67)	317.86 (275.54 - 369.1)	14.25 (12.48 - 16.3)	-0.057 (-0.06 - -0.055)	1265.83 (941.32 - 1583.48)	160.8 (119.79 - 201.94)	2902.9 (2137.29 - 3658.59)	158.86 (118.08 - 198.93)	-0.05 (-0.057 - -0.043)
Palestine	2.06 (1.78 - 2.41)	11.65 (10.22 - 13.42)	5.67 (4.92 - 6.62)	11.61 (10.19 - 13.34)	-0.011 (-0.015 - -0.007)	14.85 (10.96 - 18.83)	112.02 (83.2 - 139.9)	45.59 (33.88 - 58.24)	112.87 (84.08 - 142.16)	0.008 (-0.004 - 0.02)
Panama	3.01 (2.64 -	12.4 (10.93 -	4.96 (4.35 -	12.5 (10.98 -	0.026 (0.02 -	26.02 (19.4 -	127.02 (94.59 -	52.29 (38.4 -	130.66 (95.88 -	0.094 (0.083 -

	3.51)	14.28)	5.68)	14.33)	0.032)	32.87)	159.33)	66.23)	165.52)	0.104)
Papua New Guinea	5.59 (4.87 - 6.43)	14.4 (12.72 - 16.4)	13.51 (11.78 - 15.7)	14.48 (12.78 - 16.62)	0.018 (0.009 - 0.026)	40.7 (29.6 - 51.04)	126.81 (93.63 - 159.28)	104.05 (76.57 - 131.36)	129.47 (95.8 - 162.13)	0.034 (0.012 - 0.056)
Paraguay	4.59 (3.99 - 5.36)	12.28 (10.82 - 14.11)	8.97 (7.77 - 10.36)	12.3 (10.75 - 14.1)	-0.002 (-0.007 - 0.003)	38.5 (28.43 - 48.55)	122.81 (91.32 - 152.69)	82.01 (60.87 - 103.13)	124.3 (92.16 - 155.9)	0.019 (0.004 - 0.033)
Peru	25.84 (22.43 - 30.07)	12.26 (10.74 - 14.08)	41.73 (36.61 - 48.04)	12.32 (10.81 - 14.14)	0.013 (0.005 - 0.02)	214.8 (158.2 - 272.67)	124.03 (91.74 - 156.05)	419.32 (311.47 - 528.77)	127.06 (94.74 - 160.05)	0.096 (0.081 - 0.11)
Philippines	86.82 (75.4 - 100.03)	14.09 (12.42 - 16.02)	155.03 (135.62 - 179.11)	14.42 (12.7 - 16.54)	0.079 (0.072 - 0.085)	674.75 (496.7 - 843.12)	133.33 (98.86 - 165.45)	1373.69 (1017.96 - 1719.68)	139.42 (103.48 - 173.87)	0.156 (0.143 - 0.168)
Poland	42.9 (37.64 - 49.36)	11.12 (9.73 - 12.85)	42.34 (37.13 - 48.51)	11.3 (9.81 - 13.03)	0.057 (0.053 - 0.061)	506.25 (382.25 - 632.91)	119.11 (89.89 - 149.05)	592.75 (438.73 - 737)	123.72 (91.38 - 154.84)	0.137 (0.133 - 0.141)
Portugal	13.84 (12.25 - 15.68)	13.44 (11.87 - 15.35)	13.42 (11.73 - 15.13)	13.43 (11.7 - 15.4)	-0.008 (-0.016 - 0)	162.5 (122.14 - 201.25)	144.19 (108.13 - 179.1)	196.31 (147.14 - 244.1)	145.66 (109.34 - 181.6)	0.016 (0.008 - 0.025)
Puerto Rico	4.49 (3.93 - 5.19)	12.29 (10.76 - 14.23)	4.39 (3.87 - 5)	12.34 (10.79 - 14.3)	0.002 (-0.003 - 0.007)	46.2 (34.69 - 58.43)	127.57 (95.66 - 161.37)	55.76 (41.33 - 69.02)	129.06 (95.67 - 161.44)	0.042 (0.033 - 0.051)
Qatar	0.68 (0.59 - 0.81)	12.26 (10.72 - 14.22)	5.11 (4.31 - 6.17)	12.36 (10.76 - 14.24)	0.046 (0.039 - 0.054)	6.31 (4.58 - 8.14)	125.22 (92.99 - 157.27)	49.42 (36.19 - 63.94)	128.64 (96.15 - 162.27)	0.127 (0.113 - 0.141)
Romania	25.64 (22.42 - 29.22)	11.09 (9.66 - 12.76)	19.78 (17.47 - 22.4)	11.21 (9.82 - 12.93)	0.044 (0.035 - 0.054)	296.95 (221.29 - 370.9)	117.69 (87.28 - 146.96)	289.63 (212.78 - 363.19)	121.66 (88.78 - 153.97)	0.137 (0.124 - 0.15)
Russian Federation	159.37 (140.58 - 183.5)	10.34 (9.12 - 11.94)	150.7 (133.08 - 172.63)	10.61 (9.34 - 12.26)	0.132 (0.086 - 0.177)	1945.81 (1460.18 - 2407.57)	113.95 (85.47 - 141.38)	2126.17 (1590.07 - 2609.96)	117.6 (87.29 - 145.21)	0.154 (0.092 - 0.216)
Rwanda	7.13 (6.21 - 8.23)	11.7 (10.38 - 13.29)	13.75 (12.12 - 15.86)	11.72 (10.37 - 13.31)	0.005 (-0.002 - 0.012)	48.8 (36.44 - 61.68)	99.03 (74.57 - 124.26)	99.27 (73.26 - 125.27)	101.1 (75.6 - 126.27)	0.127 (0.067 - 0.187)
Saint Lucia	0.16 (0.14 -	12.08 (10.63 -	0.23 (0.2 -	12.06 (10.59 -	-0.016 (-0.023	1.3 (0.96 -	121.37 (89.26 -	2.5 (1.84 -	122.88 (89.85 -	0.029 (0.021 -

	0.19)	13.81)	0.26)	13.84)	- -0.009)	1.66)	154.17)	3.12)	153.23)	0.038)
Saint Vincent and the Grenadines	0.13 (0.11 - 0.15)	11.98 (10.54 - 13.72)	0.14 (0.12 - 0.16)	12.01 (10.52 - 13.73)	-0.004 (-0.012 - 0.004)	1.02 (0.76 - 1.28)	118.6 (89.12 - 148.01)	1.51 (1.11 - 1.88)	120.85 (89.06 - 150.22)	0.066 (0.056 - 0.075)
Samoa	0.24 (0.2 - 0.28)	15.04 (13.23 - 17.21)	0.28 (0.24 - 0.32)	15.11 (13.28 - 17.32)	0.038 (0.031 - 0.046)	1.77 (1.32 - 2.23)	143.7 (107.22 - 179.88)	2.45 (1.83 - 3.04)	145.56 (108.78 - 180.44)	0.085 (0.07 - 0.101)
Sao Tome and Principe	0.12 (0.1 - 0.14)	12.15 (10.74 - 13.82)	0.24 (0.21 - 0.27)	12.16 (10.72 - 13.87)	0.008 (0.005 - 0.011)	0.88 (0.65 - 1.11)	109.71 (81.62 - 137.5)	1.85 (1.37 - 2.33)	111.18 (82.85 - 138.88)	0.062 (0.047 - 0.076)
Saudi Arabia	19.1 (16.48 - 22.25)	12.09 (10.56 - 13.93)	53.52 (46.38 - 62.37)	12.09 (10.54 - 13.9)	0.01 (0.003 - 0.017)	153.84 (112.35 - 194.21)	123.36 (90.9 - 153.55)	517.36 (380.37 - 662.87)	124.89 (93.29 - 157.58)	0.045 (0.036 - 0.053)
Senegal	7.6 (6.6 - 8.75)	12.09 (10.62 - 13.73)	16.32 (14.15 - 18.74)	12.12 (10.62 - 13.83)	0.005 (-0.001 - 0.011)	54.03 (39.63 - 67.77)	106.61 (79.65 - 132.92)	121.21 (89.26 - 152.39)	108.67 (81.29 - 136.4)	0.066 (0.055 - 0.077)
Serbia	10.42 (9.17 - 11.97)	11.12 (9.68 - 12.86)	9.43 (8.28 - 10.81)	11.17 (9.71 - 12.95)	0.02 (0.014 - 0.027)	126.81 (93.4 - 159.38)	119.33 (88.03 - 150.42)	126.9 (95.03 - 158.1)	120.62 (90.04 - 150.68)	0.053 (0.037 - 0.069)
Seychelles	0.11 (0.1 - 0.13)	14.53 (12.76 - 16.67)	0.16 (0.14 - 0.18)	14.99 (13.16 - 17.19)	0.112 (0.106 - 0.117)	0.95 (0.7 - 1.2)	144.57 (106.86 - 181.14)	1.78 (1.31 - 2.24)	151.82 (111.65 - 191.38)	0.171 (0.159 - 0.182)
Sierra Leone	3.96 (3.46 - 4.56)	12.01 (10.6 - 13.59)	8.81 (7.71 - 10.14)	11.98 (10.62 - 13.51)	-0.011 (-0.021 - -0.001)	28.7 (21.36 - 36.2)	102.94 (77.06 - 128.23)	61.66 (45.77 - 77.53)	103.41 (77.63 - 129.75)	-0.014 (-0.056 - 0.029)
Singapore	5.4 (4.69 - 6.27)	13.76 (12.06 - 15.78)	8.94 (7.82 - 10.3)	13.86 (12.13 - 15.89)	0.02 (0 - 0.041)	55.75 (40.7 - 70.54)	156.11 (115.08 - 196.38)	113.75 (85.33 - 142.29)	160.08 (119.56 - 200.7)	0.091 (0.075 - 0.106)
Slovakia	5.94 (5.18 - 6.86)	11.21 (9.77 - 13)	6.1 (5.37 - 7.04)	11.35 (9.94 - 13.16)	0.055 (0.048 - 0.062)	68.45 (51.2 - 85.71)	121.11 (90.5 - 152.07)	85.02 (63.39 - 105.89)	124.44 (92.46 - 155.93)	0.113 (0.107 - 0.119)
Slovenia	2.35 (2.05 - 2.71)	11.34 (9.89 - 13.23)	2.12 (1.87 - 2.42)	11.42 (9.95 - 13.26)	0.039 (0.032 - 0.047)	28.14 (20.94 - 35.07)	123.31 (91.45 - 154.74)	32.24 (24.08 - 39.82)	125.13 (93.24 - 157.37)	0.067 (0.06 - 0.074)
Solomon Islands	0.44 (0.38 - 0.51)	14.53 (12.86 - 16.64)	0.89 (0.77 - 1.02)	14.49 (12.75 - 16.59)	-0.01 (-0.014 - -0.006)	3.09 (2.26 - 3.89)	131.29 (96.73 - 163.5)	6.98 (5.03 - 8.77)	131.34 (95.38 - 164.07)	-0.031 (-0.048 - -0.015)

Somalia	6.69 (5.88 - 7.63)	11.69 (10.34 - 13.19)	16.7 (14.63 - 19.17)	11.65 (10.28 - 13.14)	-0.019 (-0.025 - -0.012)	48.09 (35.61 - 61.08)	96.75 (71.76 - 121.55)	112.9 (83.37 - 142.2)	95.89 (70.98 - 119.64)	-0.03 (-0.051 - -0.009)
South Africa	43.89 (38.32 - 50.55)	11.9 (10.48 - 13.56)	71.22 (62.61 - 81.87)	11.91 (10.54 - 13.55)	0.006 (-0.003 - 0.014)	338.64 (250.12 - 423.42)	109.8 (82.07 - 136.17)	625.16 (470.51 - 779.63)	110.25 (83.27 - 136.58)	0.012 (-0.016 - 0.041)
South Korea	71.45 (62.31 - 83.08)	13.8 (12.08 - 15.78)	75.22 (66.47 - 85.11)	13.93 (12.23 - 16.06)	0.035 (0.02 - 0.05)	698.26 (513.1 - 873.08)	152.48 (112.42 - 190.02)	1079.54 (800.2 - 1339.79)	156.41 (115.54 - 194.87)	0.106 (0.092 - 0.121)
South Sudan	6.26 (5.43 - 7.28)	11.88 (10.49 - 13.51)	9.84 (8.6 - 11.28)	11.85 (10.46 - 13.49)	-0.015 (-0.023 - -0.006)	42.82 (31.22 - 54.52)	103.44 (76.58 - 129.95)	71.16 (52.57 - 89.3)	103.61 (76.94 - 128.8)	0.024 (0.012 - 0.035)
Spain	58.57 (54.4 - 62.91)	14.29 (13.25 - 15.36)	61.79 (55.88 - 67.14)	14.44 (13.3 - 15.57)	0.009 (-0.005 - 0.024)	716.12 (542.28 - 859.08)	163.44 (123.07 - 196.68)	974.28 (737.33 - 1169.18)	165.03 (125.49 - 197.29)	0.022 (0.017 - 0.028)
Sri Lanka	25.68 (22.42 - 29.68)	14.23 (12.53 - 16.18)	32.17 (28.49 - 36.52)	14.68 (12.91 - 16.79)	0.108 (0.102 - 0.114)	221.14 (162.38 - 277.1)	136.87 (100.98 - 170.57)	346.32 (258.83 - 430.13)	146.37 (109.51 - 183.37)	0.249 (0.241 - 0.257)
Sudan	20.24 (17.66 - 23.42)	11.32 (9.99 - 12.98)	43.47 (37.8 - 50.63)	11.35 (9.94 - 13.06)	0.018 (0.013 - 0.022)	151.57 (110.66 - 190.05)	105.11 (77.76 - 131.44)	336.21 (248.92 - 426.5)	108.03 (80.85 - 135.14)	0.118 (0.104 - 0.132)
Suriname	0.45 (0.41 - 0.5)	11.23 (10.21 - 12.5)	0.66 (0.6 - 0.72)	11.2 (10.21 - 12.35)	-0.01 (-0.016 - -0.005)	3.85 (2.86 - 4.76)	114.57 (85.5 - 141.5)	7.04 (5.24 - 8.67)	115.31 (85.89 - 142.1)	0.042 (0.028 - 0.057)
Swaziland	0.79 (0.68 - 0.91)	11.65 (10.26 - 13.25)	1.33 (1.17 - 1.54)	11.7 (10.32 - 13.28)	0.012 (0.009 - 0.015)	5.41 (3.96 - 6.88)	102.73 (75.98 - 128.43)	9.73 (7.09 - 12.3)	103.89 (76.29 - 129.62)	-0.01 (-0.037 - 0.017)
Sweden	12.34 (10.95 - 13.84)	14.58 (12.96 - 16.5)	13.33 (11.82 - 15.12)	14.17 (12.46 - 16.17)	-0.09 (-0.107 - -0.073)	166.52 (125.15 - 206.09)	164.92 (123.27 - 205.04)	189.57 (142.77 - 234.26)	158.45 (118.67 - 196.83)	-0.119 (-0.143 - -0.094)
Switzerland	10.46 (9.19 - 12.03)	13.87 (12.19 - 15.94)	11.6 (10.24 - 13.14)	13.79 (12.17 - 15.84)	-0.026 (-0.033 - -0.02)	128.58 (97.24 - 159.95)	152.87 (115.19 - 190.61)	165.01 (123.8 - 203.88)	152.36 (113.03 - 189.86)	-0.014 (-0.023 - -0.005)
Syria	13.06 (11.2 - 15.22)	11.65 (10.19 - 13.37)	19.69 (17.22 - 22.53)	11.63 (10.23 - 13.4)	0.017 (0.009 - 0.024)	97.11 (71.47 - 122.91)	113.31 (83.4 - 141.39)	185.75 (138.45 - 233.45)	114.24 (84.88 - 143.66)	0.089 (0.067 - 0.112)
Tajikistan	33.6 (29.66 - 38.79)	14.89 (13.22 - 16.99)	38.08 (33.76 - 43.09)	15.46 (13.64 - 17.75)	0.144 (0.128 - 0.159)	311.34 (232.45 - 387.89)	147.45 (110.77 - 182.28)	476.45 (353.67 - 593.44)	157.5 (117.79 - 197.58)	0.246 (0.231 - 0.261)

Tanzania	5.36 (4.61 - 6.31)	10.64 (9.31 - 12.3)	10.32 (8.94 - 12.16)	10.56 (9.27 - 12.26)	-0.019 (-0.032 - -0.006)	44.73 (32.93 - 56.42)	114.29 (84.66 - 143.42)	93.53 (68.26 - 118.63)	112.24 (83.06 - 141.3)	-0.054 (-0.1 - -0.007)
Thailand	25.33 (22.06 - 29.1)	11.72 (10.36 - 13.23)	56.11 (48.84 - 64.28)	11.75 (10.37 - 13.34)	0.014 (0.005 - 0.022)	172.93 (128.89 - 220.19)	99.77 (74.88 - 125.44)	407.22 (296.95 - 515.81)	103.45 (75.94 - 131.21)	0.167 (0.134 - 0.201)
The Bahamas	89.17 (77.1 - 102.46)	14.13 (12.37 - 16.06)	108.82 (96.37 - 121.99)	14.62 (12.88 - 16.69)	0.124 (0.117 - 0.131)	751.21 (544.31 - 951.77)	136.01 (99.34 - 170.31)	1278.62 (954.7 - 1586.39)	145.66 (108.82 - 181.83)	0.245 (0.234 - 0.256)
The Gambia	0.35 (0.3 - 0.4)	12.21 (10.71 - 13.98)	0.49 (0.43 - 0.56)	12.11 (10.61 - 13.96)	-0.018 (-0.023 - -0.013)	2.95 (2.19 - 3.72)	125.38 (92.69 - 157.07)	5.3 (3.92 - 6.69)	124.76 (92.57 - 158.2)	0 (-0.012 - 0.012)
Timor-Leste	1.02 (0.88 - 1.19)	12.05 (10.61 - 13.71)	2.38 (2.07 - 2.75)	12.05 (10.63 - 13.72)	0.001 (-0.004 - 0.006)	6.99 (5.12 - 8.88)	104.65 (77.3 - 131.18)	16.62 (12.12 - 20.99)	104.97 (77.24 - 132.2)	0.017 (0.009 - 0.024)
Togo	0.98 (0.85 - 1.13)	13.2 (11.62 - 14.97)	1.68 (1.46 - 1.94)	13.58 (11.91 - 15.47)	0.121 (0.114 - 0.127)	7.28 (5.31 - 9.34)	117.92 (86.81 - 148.36)	12.92 (9.59 - 16.26)	127.08 (94.26 - 158.67)	0.31 (0.286 - 0.334)
Tonga	3.66 (3.18 - 4.21)	11.96 (10.57 - 13.66)	8.45 (7.38 - 9.72)	11.93 (10.55 - 13.56)	-0.006 (-0.012 - 0.001)	24.57 (17.85 - 31.18)	102.57 (75.24 - 128.6)	62.78 (45.96 - 79.49)	103.19 (75.72 - 129.07)	0.01 (-0.006 - 0.026)
Trinidad and Tobago	0.14 (0.12 - 0.16)	15.05 (13.33 - 17.06)	0.15 (0.13 - 0.17)	15.1 (13.33 - 17.28)	0.031 (0.025 - 0.037)	1.05 (0.78 - 1.31)	143.46 (106.27 - 178.23)	1.33 (0.99 - 1.67)	145.1 (108.04 - 180.97)	0.052 (0.043 - 0.061)
Tunisia	1.23 (1.14 - 1.34)	9.96 (9.32 - 10.83)	1.46 (1.36 - 1.59)	10 (9.34 - 10.97)	0.017 (0.012 - 0.022)	11.37 (8.51 - 14)	104.49 (78.53 - 128.25)	17.68 (13.27 - 21.77)	107.05 (80.32 - 131.92)	0.12 (0.106 - 0.135)
Turkey	9.83 (8.57 - 11.46)	11.8 (10.41 - 13.5)	14.29 (12.6 - 16.4)	11.8 (10.34 - 13.56)	0.011 (0.006 - 0.017)	81.27 (59.64 - 102.31)	117.03 (86.34 - 146.51)	154.3 (112.67 - 193.53)	119.14 (87.09 - 149.72)	0.077 (0.069 - 0.086)
Turkmenistan	75.28 (65.18 - 86.72)	12.83 (11.25 - 14.6)	108.76 (95.54 - 122.28)	12.82 (11.21 - 14.47)	-0.007 (-0.011 - -0.003)	648.28 (475 - 809.69)	129.81 (95.05 - 161.43)	1186.87 (887.63 - 1482.56)	132.61 (98.99 - 165.72)	0.068 (0.063 - 0.073)
Uganda	3.89 (3.32 - 4.58)	10.66 (9.24 - 12.34)	5.77 (5.02 - 6.74)	10.81 (9.43 - 12.52)	0.045 (0.022 - 0.067)	33.31 (24.82 - 42.33)	115.97 (86.87 - 145.94)	61.69 (45.9 - 78.39)	119.85 (89.15 - 151.31)	0.118 (0.069 - 0.168)
Ukraine	16.36 (14.21 - 18.99)	11.63 (10.28 - 13.2)	38.75 (33.66 - 44.72)	11.68 (10.36 - 13.26)	0.017 (0.011 - 0.023)	103.22 (74.94 - 130.87)	93.31 (69.07 - 116.13)	254.97 (188.62 - 323.47)	99.95 (74.81 - 125.12)	0.275 (0.259 - 0.291)

United Arab Emirates	54.58 (48.26 - 62.31)	10.51 (9.16 - 12.06)	45.91 (40.64 - 52.74)	10.44 (9.21 - 12.11)	-0.02 (-0.042 - 0.002)	685.75 (511.52 - 858.38)	114.32 (85.08 - 143.56)	637.62 (483.72 - 799.77)	113.11 (85.68 - 143.29)	-0.011 (-0.054 - 0.032)
United Kingdom	2.77 (2.38 - 3.32)	12.21 (10.59 - 14.08)	16.02 (13.54 - 18.95)	12.24 (10.7 - 14.1)	0.049 (0.035 - 0.062)	24.97 (18.14 - 31.96)	124.7 (91.89 - 155.37)	194.3 (140.6 - 247.08)	126.44 (93.27 - 159.77)	0.101 (0.08 - 0.121)
United States	71.57 (63.66 - 80.82)	12.27 (10.87 - 13.93)	81.59 (72.71 - 92.22)	12.93 (11.42 - 14.71)	0.121 (0.07 - 0.171)	972.72 (732.38 - 1194.29)	147.02 (110.46 - 180.88)	1204.27 (910.59 - 1482.03)	152.2 (114.22 - 188.23)	0.059 (0.021 - 0.096)
Uruguay	385.89 (339.98 - 441.65)	14.7 (12.92 - 16.75)	428.83 (379.08 - 484.69)	14.27 (12.55 - 16.26)	-0.069 (-0.101 - -0.036)	5549.07 (4166.39 - 6849.22)	196.73 (147.34 - 242.91)	7177.84 (5387.13 - 8803.71)	191.69 (142.86 - 236.23)	-0.038 (-0.073 - -0.004)
Uzbekistan	3.25 (2.86 - 3.69)	10.68 (9.33 - 12.19)	3.56 (3.13 - 4.03)	10.55 (9.23 - 12.05)	-0.024 (-0.033 - -0.014)	37.85 (27.77 - 47.68)	117.15 (85.85 - 147.62)	44.23 (33.55 - 54.86)	116.78 (87.53 - 145.72)	0.01 (-0.004 - 0.024)
Vanuatu	21.53 (18.61 - 25.1)	10.57 (9.26 - 12.11)	37.86 (32.98 - 44.23)	10.66 (9.35 - 12.35)	0.021 (0.014 - 0.029)	181.93 (133.75 - 230.14)	112.57 (82.67 - 140.77)	378.67 (280.86 - 475.85)	115.44 (86.12 - 144.44)	0.075 (0.05 - 0.099)
Venezuela	0.2 (0.17 - 0.23)	14.61 (12.93 - 16.57)	0.4 (0.35 - 0.46)	14.58 (12.89 - 16.59)	0.017 (0.009 - 0.024)	1.51 (1.12 - 1.9)	133.35 (98.77 - 167.38)	3.22 (2.37 - 4.04)	133.33 (99.3 - 166.65)	0.03 (0.02 - 0.041)
Vietnam	23.34 (20.27 - 27.24)	12.36 (10.87 - 14.21)	41.03 (35.89 - 47.34)	12.35 (10.85 - 14.19)	-0.004 (-0.008 - 0.001)	196.57 (142.59 - 250.44)	127.21 (93.63 - 159.36)	415.1 (307.41 - 515.92)	127.2 (94.33 - 157.66)	0.009 (-0.002 - 0.02)
Virgin Islands, U.S.	101.1 (87.81 - 116.81)	14.96 (13.14 - 16.89)	161.9 (142.25 - 185.48)	15.42 (13.57 - 17.55)	0.108 (0.098 - 0.118)	791.86 (589.63 - 993.15)	142.02 (106.03 - 176.77)	1671.2 (1229.93 - 2100.79)	153.22 (112.68 - 191.87)	0.275 (0.26 - 0.289)
Yemen	0.13 (0.11 - 0.15)	12.28 (10.8 - 14.06)	0.12 (0.1 - 0.13)	12.42 (10.93 - 14.43)	0.045 (0.033 - 0.057)	1.37 (1.02 - 1.72)	127.63 (95.6 - 160.81)	1.62 (1.2 - 2.02)	131.52 (96.92 - 164.44)	0.117 (0.089 - 0.145)
Zambia	12.17 (10.53 - 14.07)	11.34 (9.95 - 13.01)	32.34 (28.23 - 37.57)	11.27 (9.95 - 12.92)	-0.006 (-0.014 - 0.002)	88.42 (65.77 - 112.03)	102.96 (77.32 - 128.67)	245 (178.47 - 307.29)	104.42 (76.94 - 130.11)	0.095 (0.072 - 0.118)
Zimbabwe	7.82 (6.78 -	11.72 (10.32 -	18.58 (16.22 -	11.75 (10.4 -	0.014 (0.002 -	51.61 (37.77 -	100.96 (74.94 -	128.81 (95.95 -	103.22 (77.79 -	0.135 (0.073 -

9.06)	13.34)	21.43)	13.39)	0.026)	65.34)	126.44)	164.93)	129.7)	0.198)

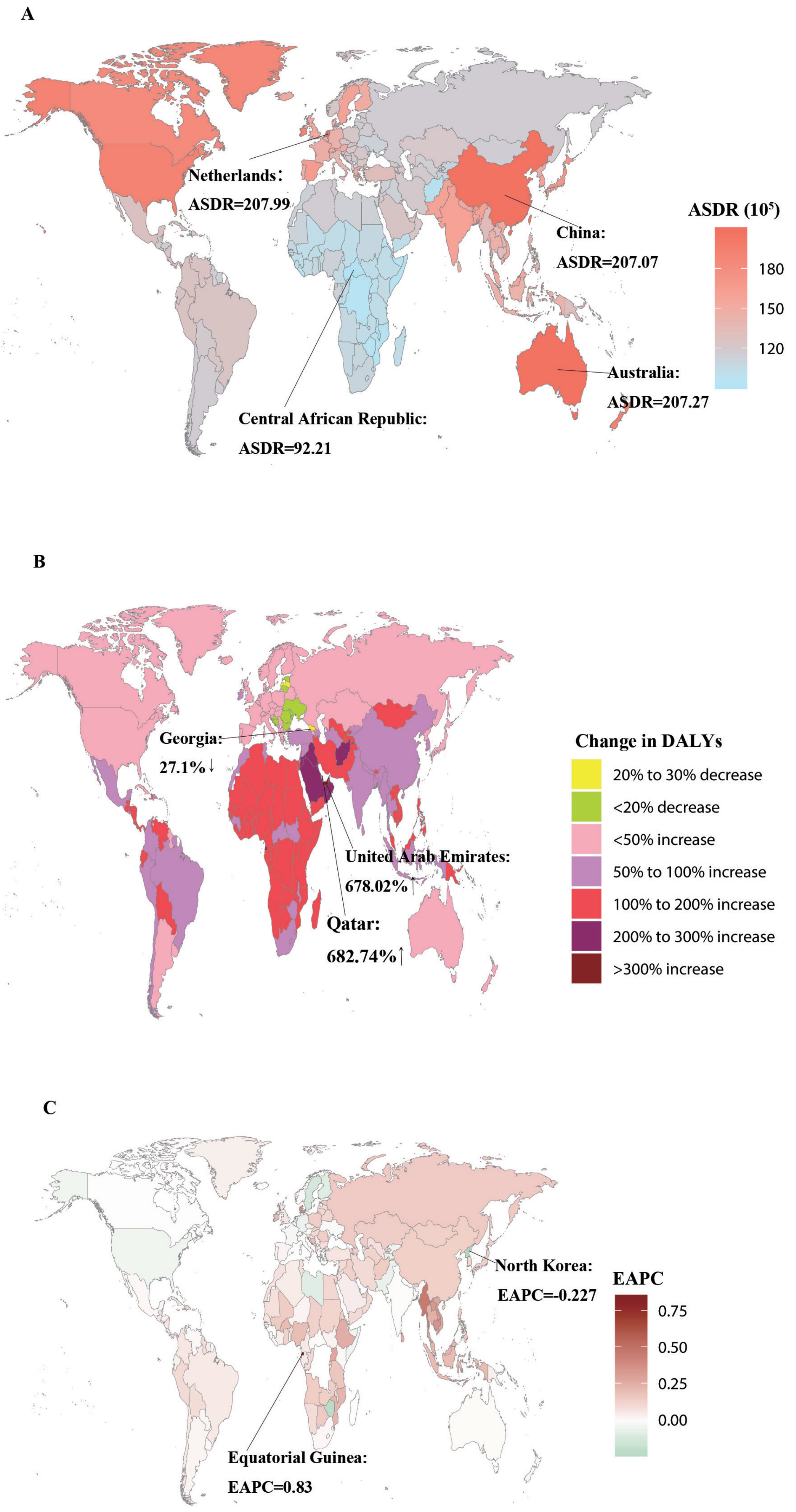
A
Netherlands：
ASDR=207.99
China:
ASDR=207.07
Australia:
ASDR=207.27
Central African Republic:
ASDR=92.21
ASDR (10⁵)
180
150
120
B
Georgia:
27.1%↓
United Arab Emirates:
678.02%↑
Qatar:
682.74%↑
Change in DALYs
20% to 30% decrease
<20% decrease
<50% increase
50% to 100% increase
100% to 200% increase
200% to 300% increase
>300% increase
C
North Korea:
EAPC=-0.227
Equatorial Guinea:
EAPC=0.83
EAPC
0.75
0.50
0.25
0.00
